# Supplementary material for: scDREAMER for atlas-level integration of single-cell datasets using deep generative model paired with adversarial classifier
Source: Nat Commun. 2023 Nov 27;14:7781. doi: 10.1038/s41467-023-43590-8 (PMC10682386; doi:10.1038/s41467-023-43590-8)
Supplement: Supplementary file 1 — Supplementary Information file [file 41467_2023_43590_MOESM1_ESM.pdf]

Supplementary Material for scDREAMER for atlas-level integration of single-cell datasets using deep generative model paired with adversarial classifier

Ajita Shree[1,†], Musale Krushna Pavan[1,†], Hamim Zafar[1,2,3,*]

[1]Department of Computer Science and Engineering,
Indian Institute of Technology Kanpur, Kanpur, India
[2]Department of Biological Sciences and Bioengineering,
Indian Institute of Technology Kanpur, Kanpur, India
[3]Mehta Family Centre for Engineering in Medicine,
Indian Institute of Technology Kanpur, Kanpur, India
[†]*Authors contributed equally*
[*]*Corresponding author, hamim@iitk.ac.in*

November 8, 2023

Contents

Supplementary Figures **2**

Supplementary Tables **26**

Supplementary Note 1: Comparison of scDREAMER against other adversarial training approaches for scRNA-seq **30**

Supplementary Figures

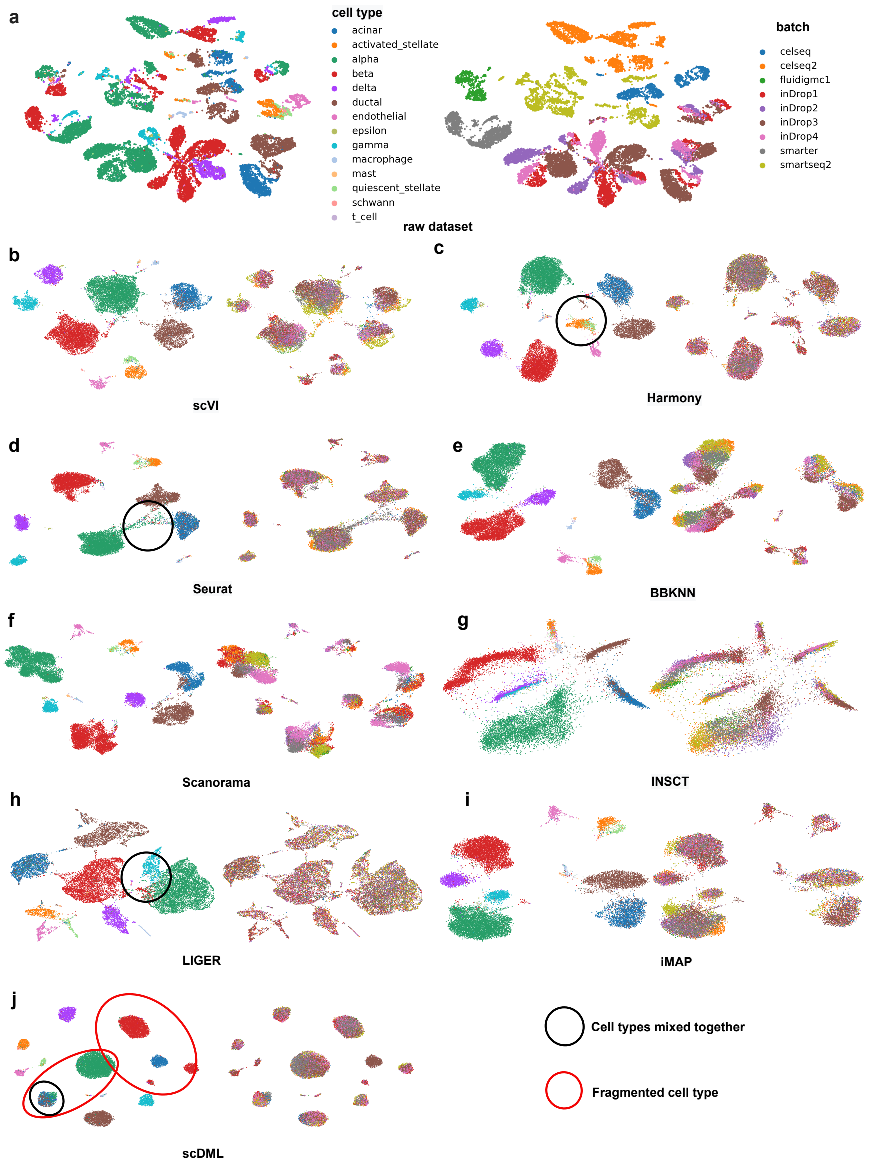

Supplementary Figure 1: Visualization of latent space embeddings for Human Pancreas Integration: a) Visualization of un-integrated human pancreas data annotated and colored by different pancreatic cell types (left) and batch information (right). The dataset is generated from different single-cell sequencing protocols. Visualization of latent space embeddings post-integration by different integration algorithms: b) scVI, c) Harmony, d) Seurat, e) BBKNN, f) Scanorama, g) INSCT, h) LIGER, i) iMAP and j) scDML.

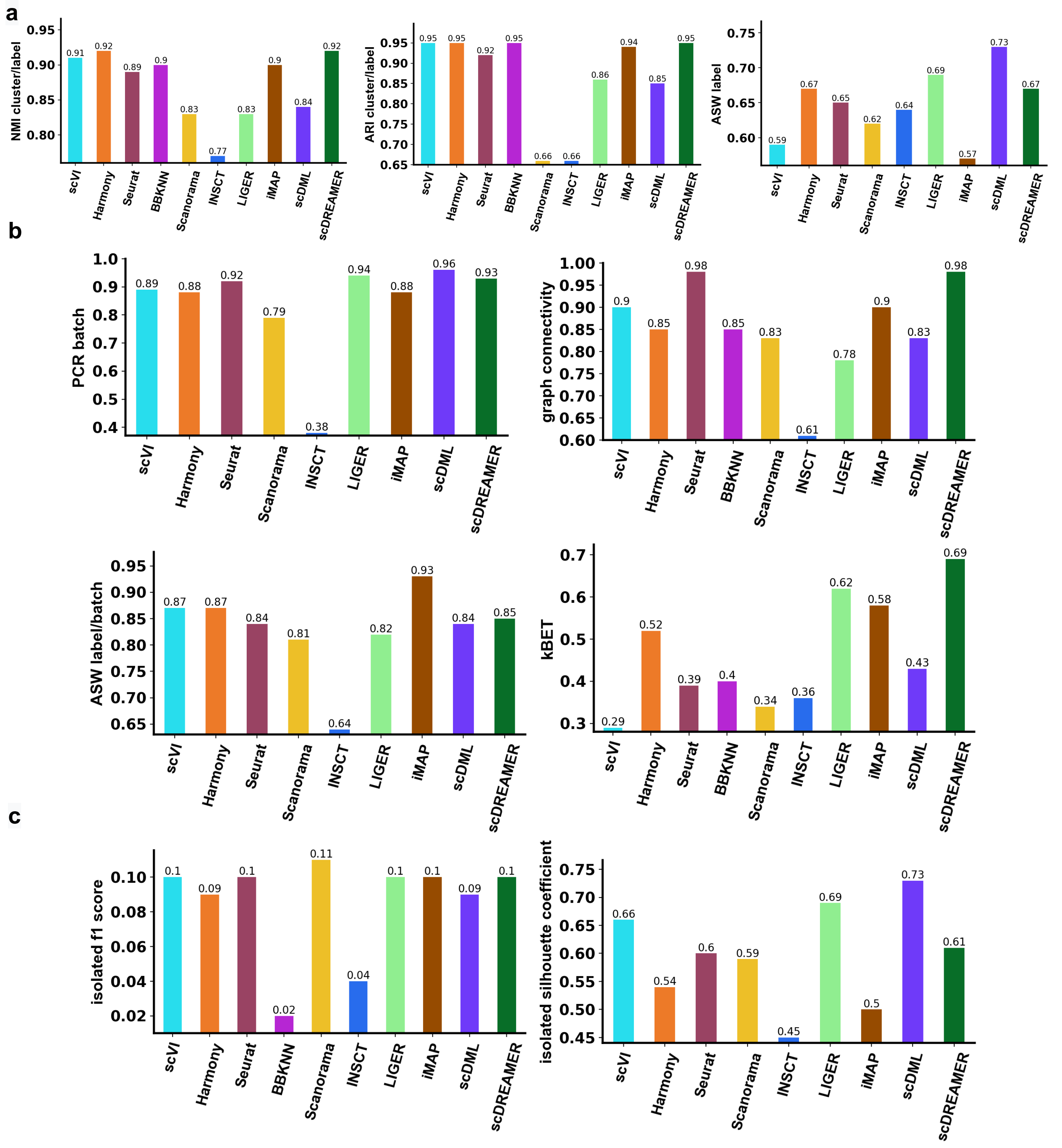

Supplementary Figure 2: Quantitative assessment of different methods for Human Pancreas Integration: a) Comparison of bio-conservation metrics i.e. NMI, ARI and ASW across different integration algorithms i.e. scVI, Harmony, Seurat, BBKNN, Scanorama, INSCT, LIGER, iMAP, scDML and scDREAMER. b) Comparison of batch-correction metrics i.e. PCR batch, graph connectivity, ASW label/batch and kBET across different integration algorithms. c) Comparison of isolated f1 score and isolated silhouette coefficient metrics across different integration algorithms. Source data are provided as a Source Data file.

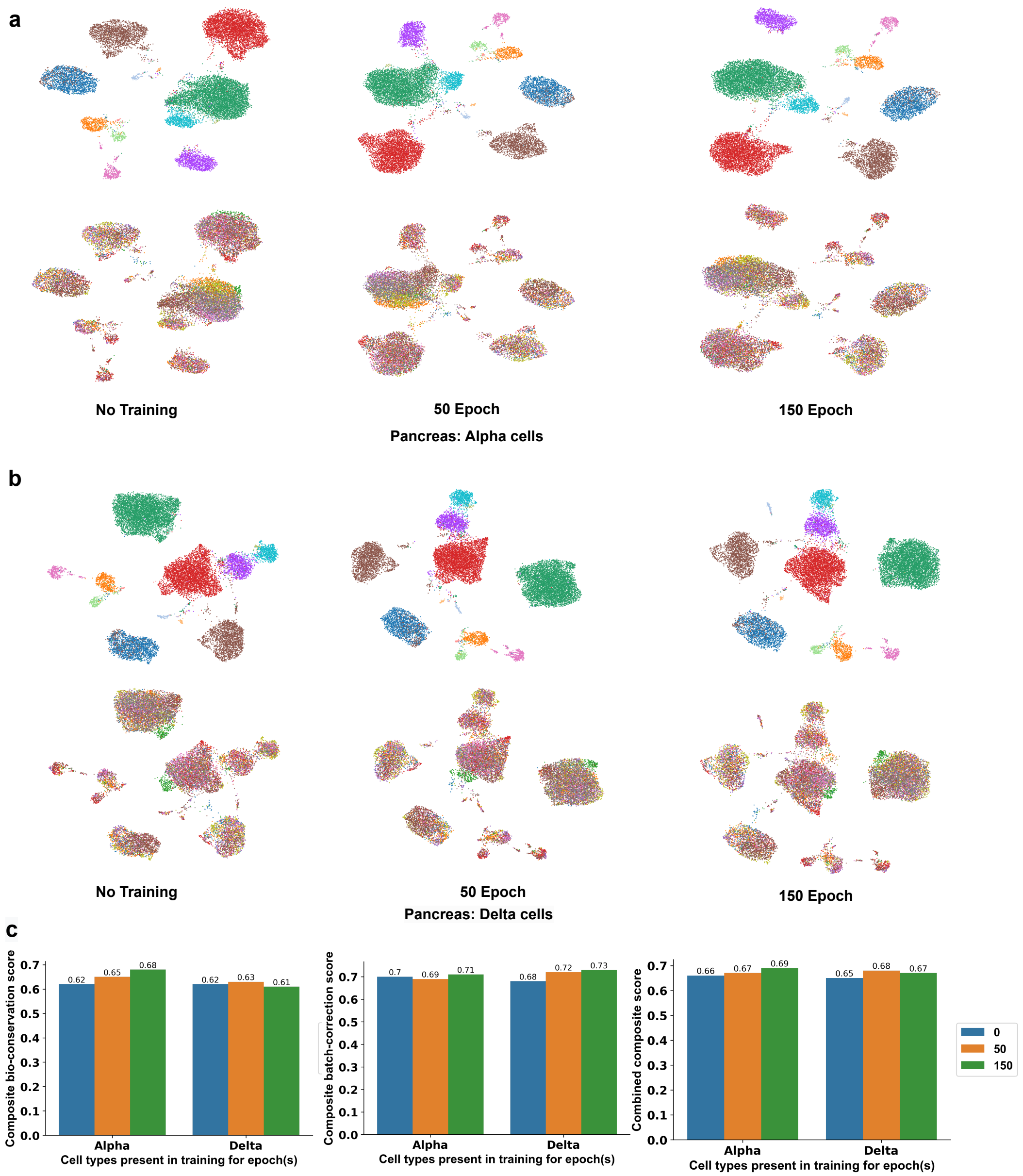

Supplementary Figure 3: Performance of scDREAMER on cell type held out during training for Pancreas integration task. a) Visualization of latent space embeddings when alpha cells are not used in training followed by scenarios when network weights get updated post-training on alpha cells for 50 and 150 epochs. b) Visualization of latent space embeddings when delta cells are not used in training followed by scenarios when network weights get updated post-training on delta cells for 50 and 150 epochs. c) Quantitative assessment of scDREAMER embeddings for the held-out cell type for three scenarios - not used in training (0 epochs) and after updating network weights post-training on the held-out cells for 50 and 150 epochs. Source data are provided as a Source Data file.

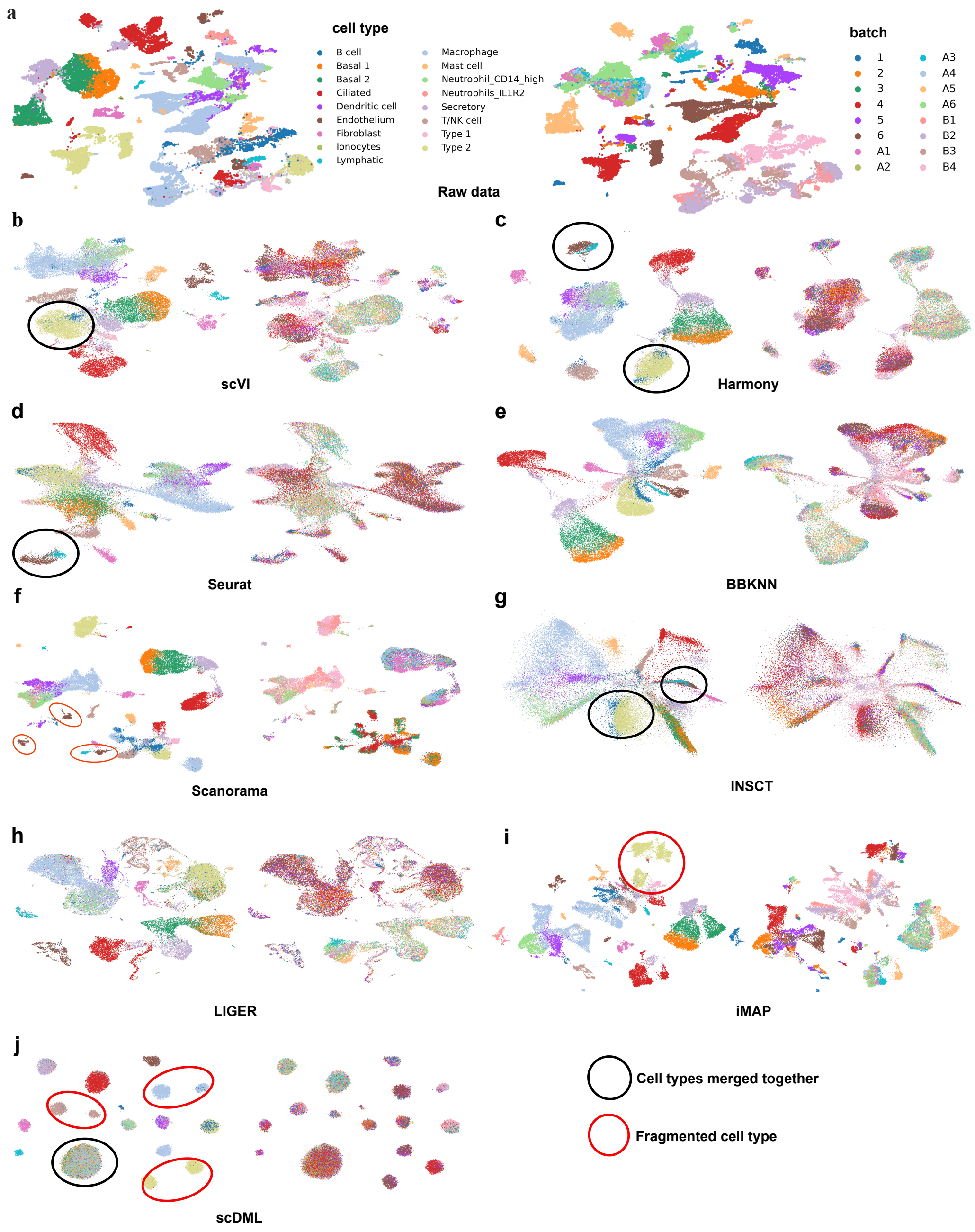

Supplementary Figure 4: Visualization of latent space embeddings for Lung Integration: a) Visualization of un-integrated Lung data annotated and coloured by different cell types (left) and batch information (right). Visualization of latent space embeddings post-integration by different integration algorithms: b) scVI, c) Harmony, d) Seurat, e) BBKNN, f) Scanorama, g) INSCT, h) LIGER i) iMAP and j) scDML.

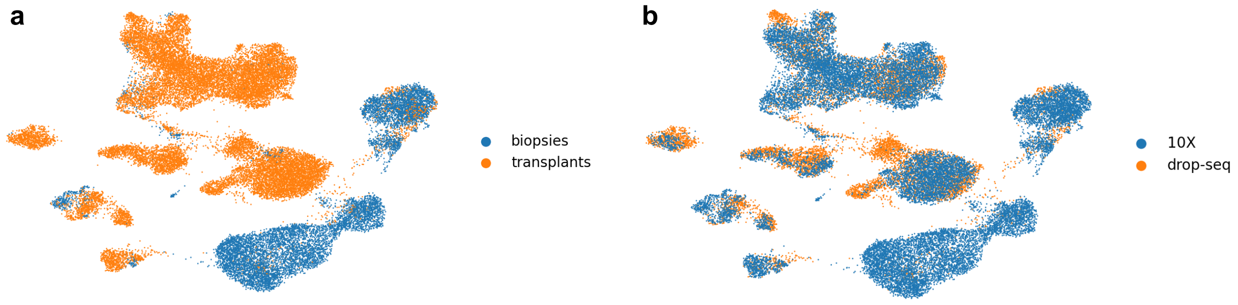

Supplementary Figure 5: Visualization of latent space embeddings inferred by scDREAMER for lung atlas integration with different annotations: a) Visualization of latent space embeddings annotated using different sampling locations. b) Visualization of latent embeddings annotated using different sequencing techniques.

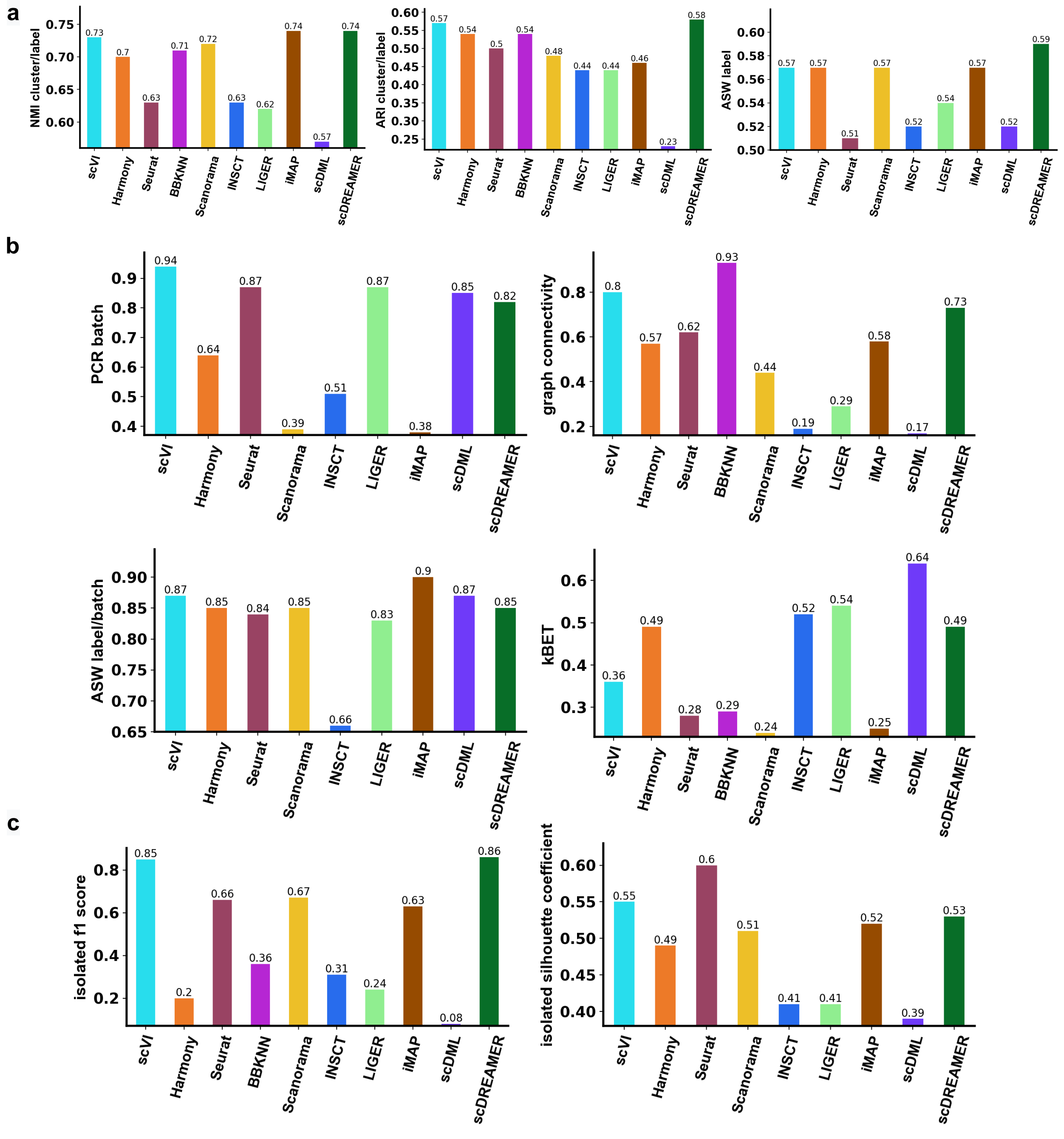

Supplementary Figure 6: Quantitative assessment of different methods for Lung Atlas Integration: a) Comparison of bio-conservation metrics i.e. NMI, ARI and ASW across different integration algorithms i.e. scVI, Harmony, Seurat, BBKNN, Scanorama, INSCT, LIGER, iMAP, scDML and scDREAMER. b) Comparison of batch-correction metrics i.e. PCR batch, graph connectivity, ASW label/batch and kBET across different integration algorithms. c) Comparison of isolated f1 score and isolated silhouette coefficient metrics across different integration algorithms. Source data are provided as a Source Data file.

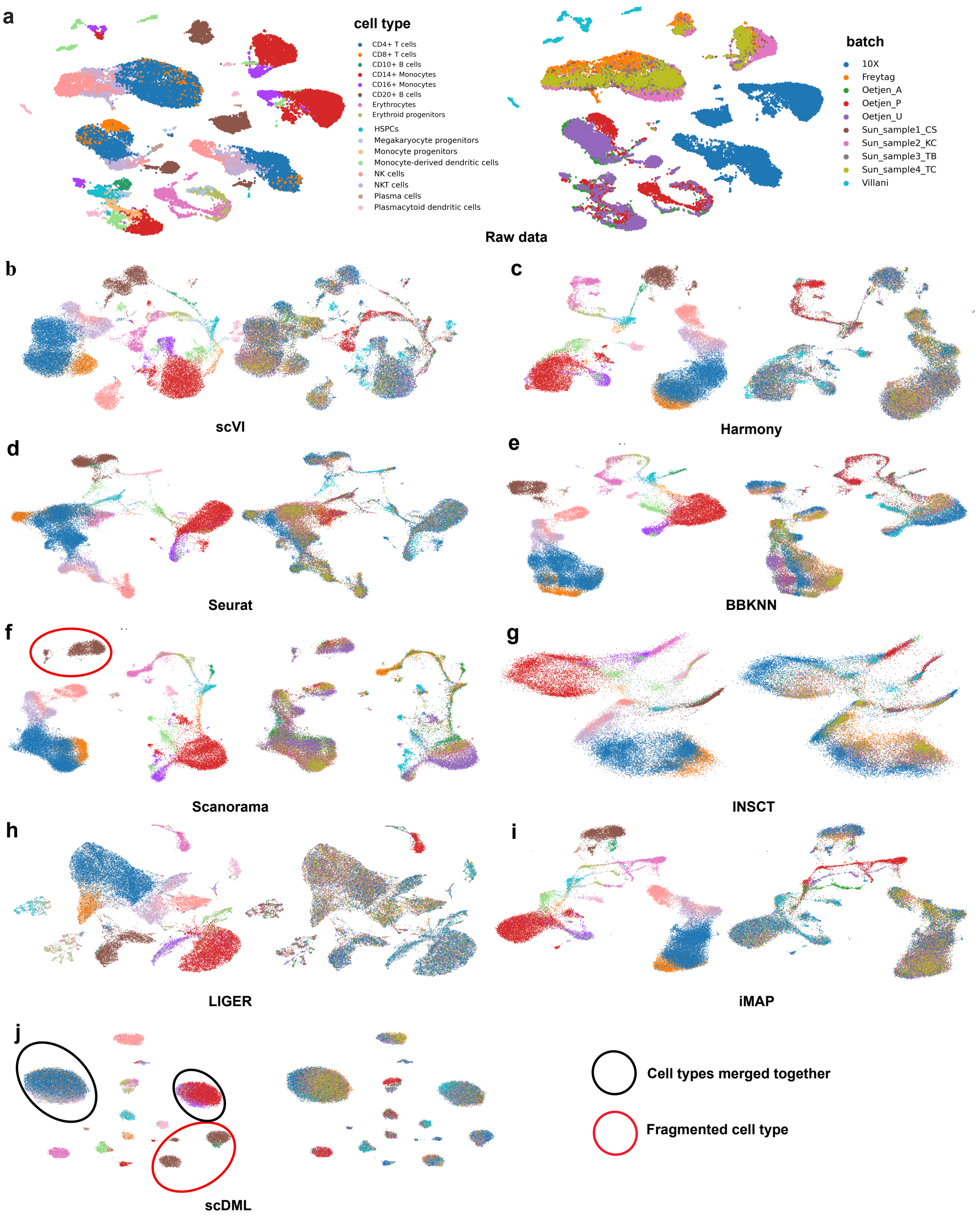

Supplementary Figure 7: Visualization of latent space embeddings for Human Immune Integration task: a) Visualization of un-integrated human immune dataset annotated and coloured by different immune cell types (left) and batch information (right). Visualization of latent space embeddings post-integration by different integration algorithms: b) scVI, c) Harmony, d) Seurat, e) BBKNN, f) Scanorama, g) INSCT, h) LIGER and i) iMAP and j) scDML .

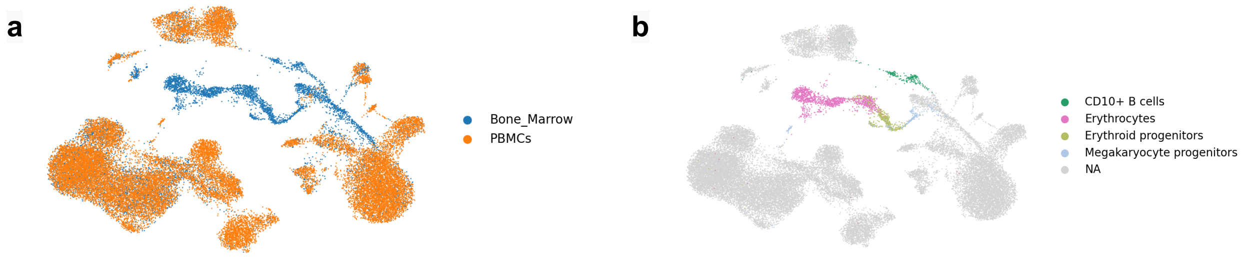

Supplementary Figure 8: Visualization of latent space embeddings inferred by scDREAMER for Human Immune Integration with different annotations: a) Visualization of Human Immune dataset annotated by tissue i.e. PBMC and Bone marrow b) Visualization highlighting the cell types in the trajectory (bone marrow specific) captured by scDREAMER.

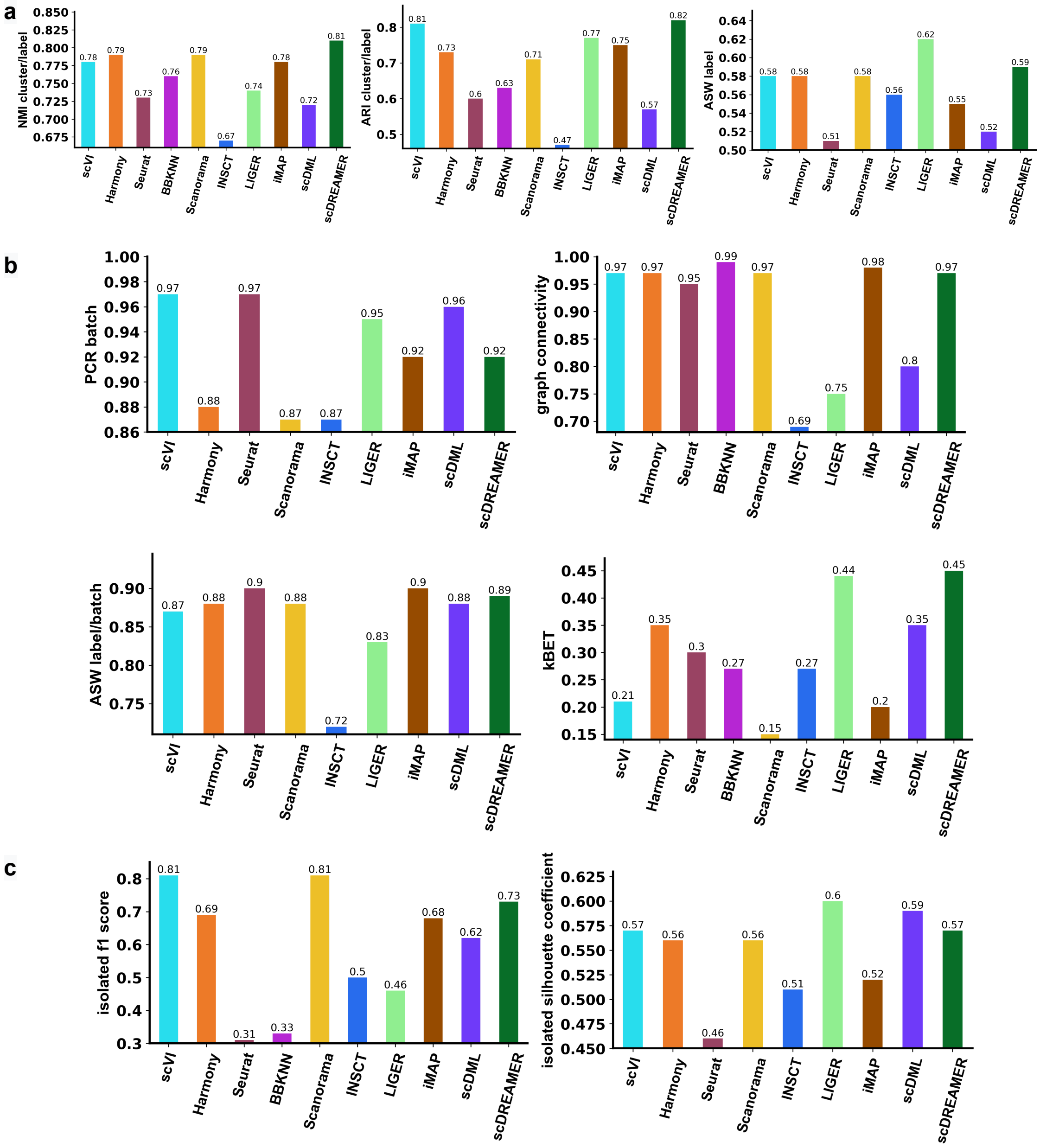

Supplementary Figure 9: Quantitative assessment of different methods for Human Immune Integration: a) Comparison of bio-conservation metrics i.e. NMI, ARI and ASW across different integration algorithms i.e. scVI, Harmony, Seurat, BBKNN, Scanorama, INSCT, LIGER, iMAP, scDML and scDREAMER. b) Comparison of batch-correction metrics i.e. PCR batch, graph connectivity, ASW label/batch and kBET across different integration algorithms. c) Comparison of isolated f1 score and isolated silhouette coefficient metrics across different integration algorithms. Source data are provided as a Source Data file.

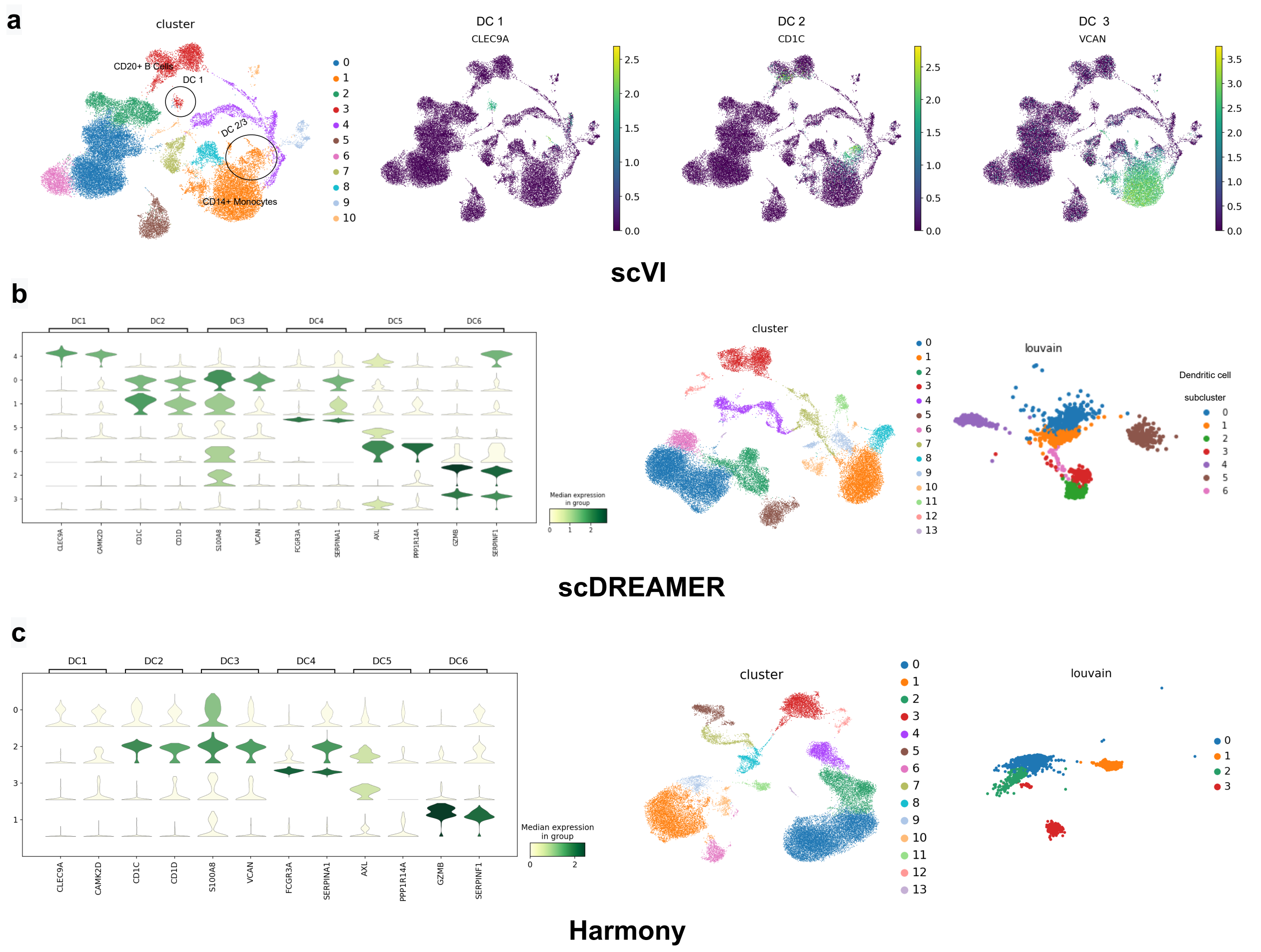

Supplementary Figure 10: Comparison of scVI, scDREAMER, and Harmony in capturing dendritic cell (DC) subtypes for human immune integration task. a) Visualization of latent space embeddings inferred by scVI for Human Immune Integration task colored by optimal clustering, and three marker genes (*CLEC9A, CD1C, VCAN*) for three dendritic cell subtypes (DC1, DC2, and DC3 respectively). b) Violin plot of marker genes for DC subtypes for the optimal clusters inferred by scDREAMER. UMAP visualization of scDREAMER-inferred embeddings colored by optimal clusters for all cells and DC subpopulations. c) Violin plot of marker genes for DC subtypes over the optimal clusters inferred by the Harmony. UMAP visualization of Harmony embeddings colored by optimal clusters for all cells and DC subpopulations respectively.

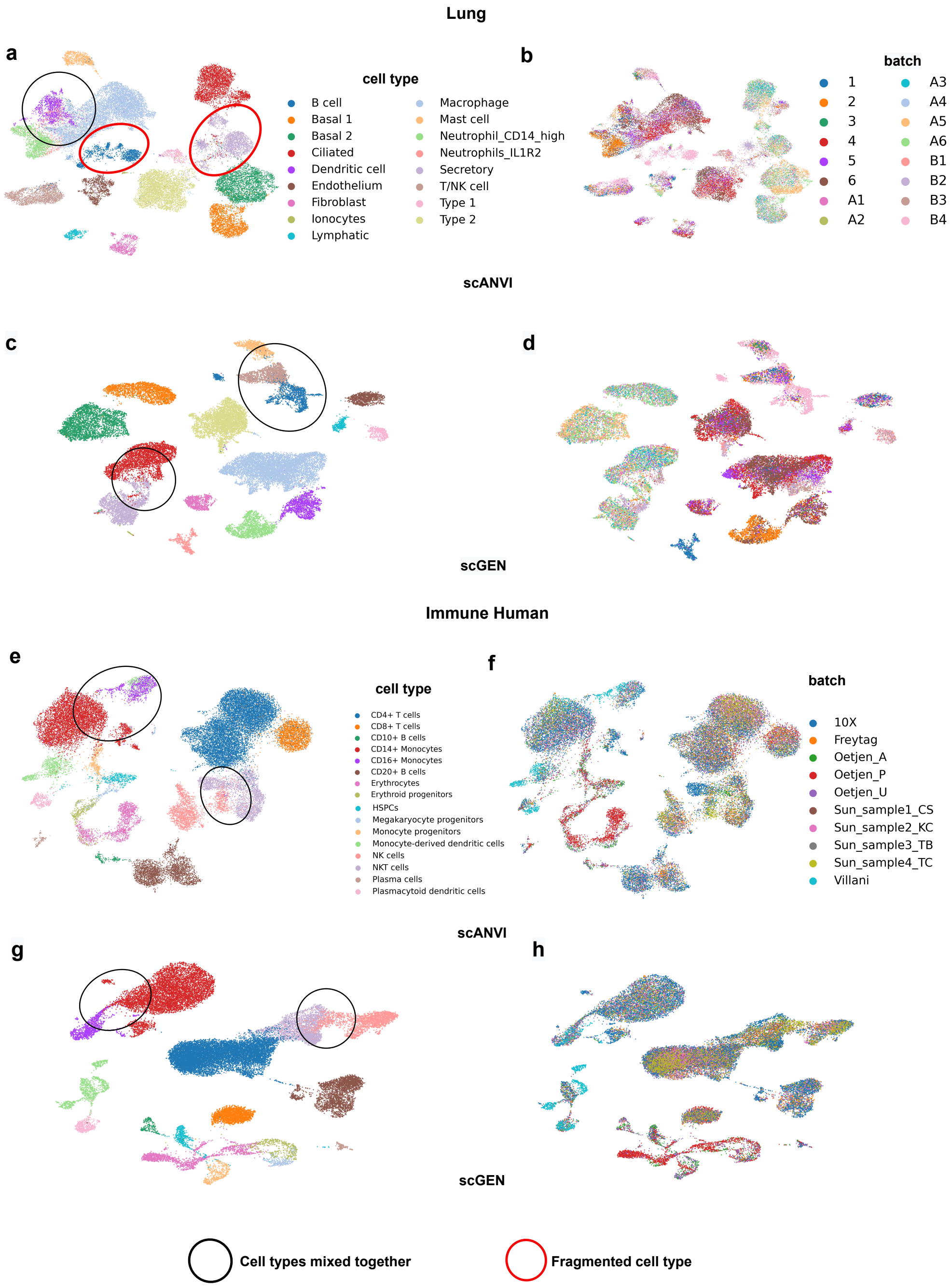

Supplementary Figure 11: Visualization of latent space embeddings inferred by supervised algorithms for Lung and Human Immune integration. a-b) Visualization of Integrated Lung data by scANVI coloured by different (a) cell types and (b) batch information. c-d) Visualization of Integrated Lung data by scGEN coloured by different (c) cell types and (d) batch information. e-f)Visualization of Integrated Human Immune data by scANVI coloured by different (e) cell types and (f) batch information. g-h) Visualization of Integrated Human Immune data by scGEN coloured by different (g) cell types and (h) batch information.

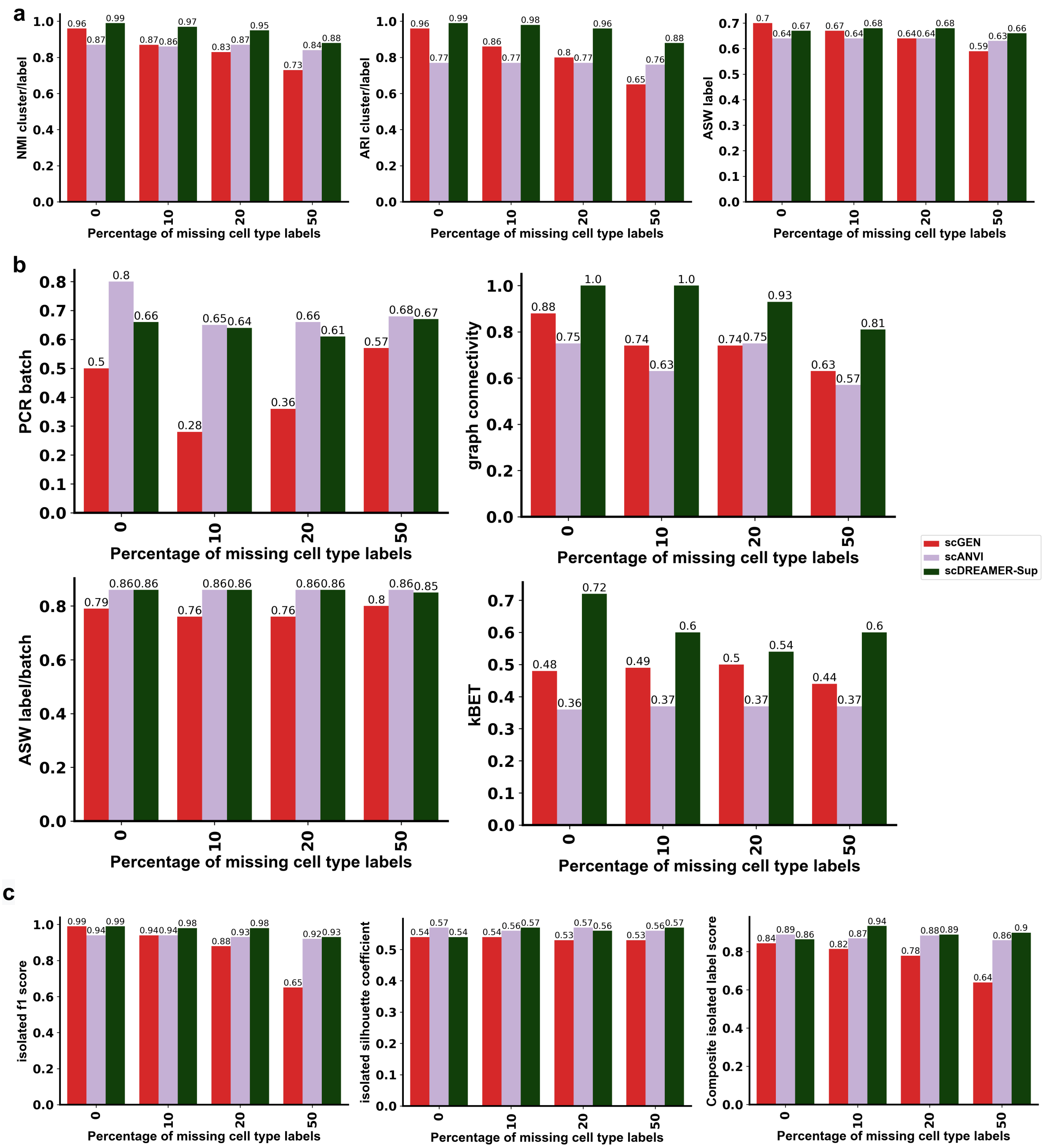

Supplementary Figure 12: Quantitative assessment of different supervised integration methods (scGEN, scANVI, and scDREAMER-Sup) for Lung Integration over different percentages of missing labels. a) Comparison of bio-conservation metrics i.e. NMI, ARI and ASW. b) Comparison of batch-correction metrics i.e. PCR batch, graph connectivity, ASW label/batch and kBET. c) Comparison of isolated f1 score, Isolated silhouette coefficient, and Composite isolated label score metrics. Source data are provided as a Source Data file.

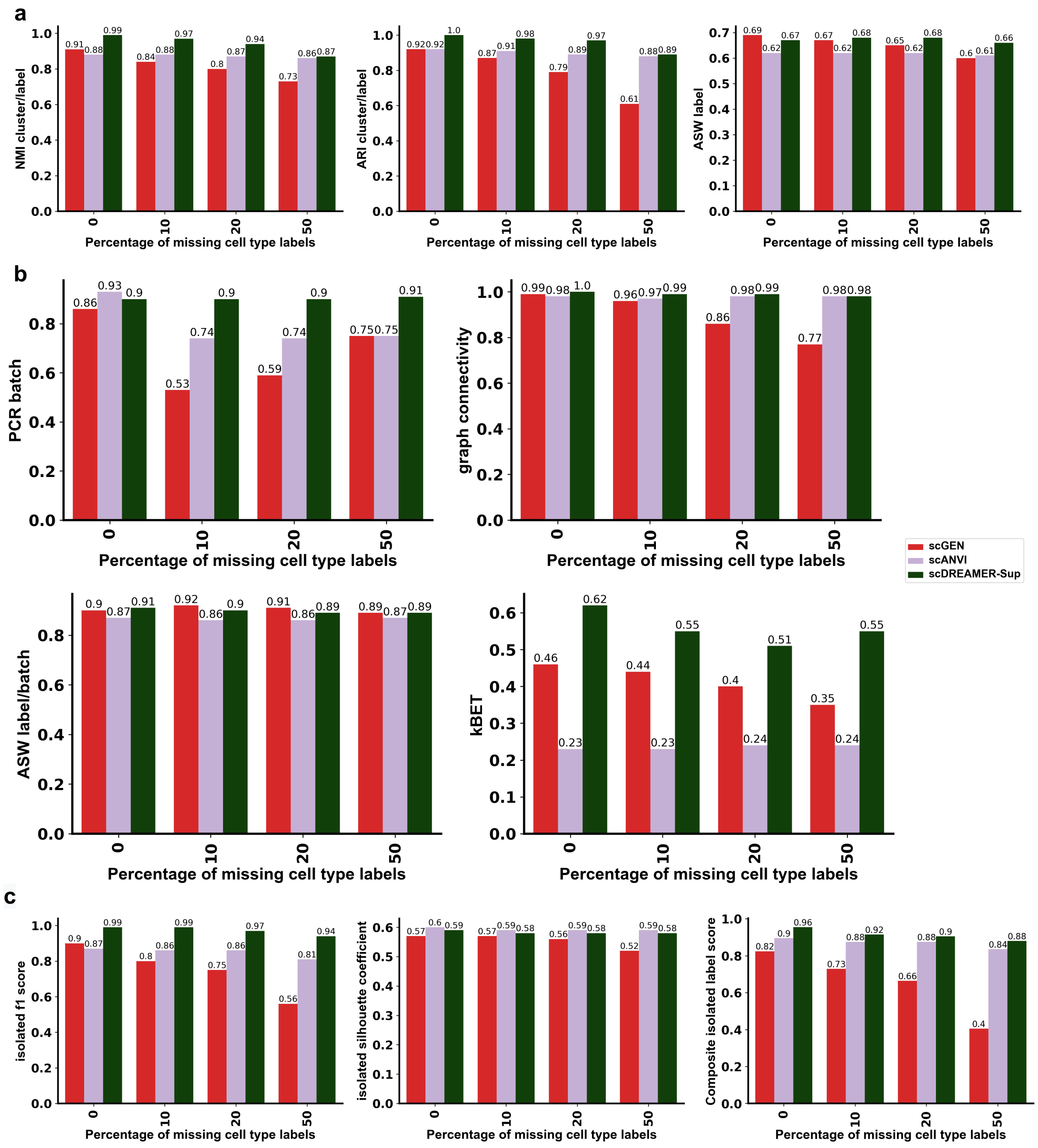

Supplementary Figure 13: Quantitative assessment of different supervised integration methods (scGEN, scANVI, and scDREAMER-Sup) for Human Immune Integration task over different percentages of missing labels. a) Comparison of bio-conservation metrics i.e. NMI, ARI and ASW. b) Comparison of batch-correction metrics i.e. PCR batch, graph connectivity, ASW label/batch, and kBET. c) Comparison of isolated f1 score, Isolated silhouette coefficient, and Composite isolated label score metrics. Source data are provided as a Source Data file.

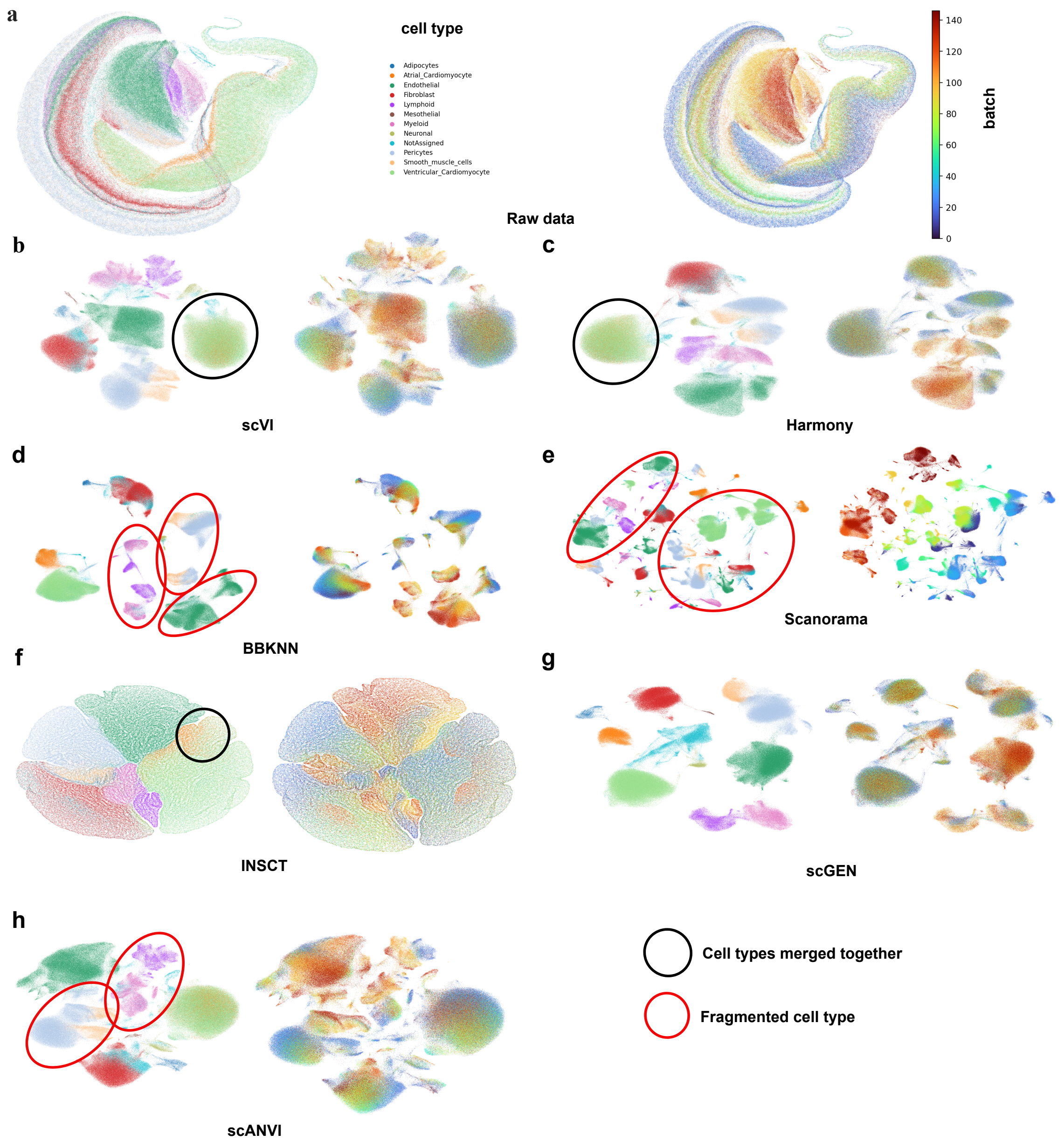

Supplementary Figure 14: Visualization of latent space embeddings for Heart Atlas Integration task: a) Visualization of un-integrated human immune dataset annotated and coloured by different immune cell types (left) and batch information (right). Visualization of latent space embeddings post-integration by different integration algorithms: b) scVI, c) Harmony, d) BBKNN, e) Scanorama, f) INSCT, g) scGEN and h) scANVI.

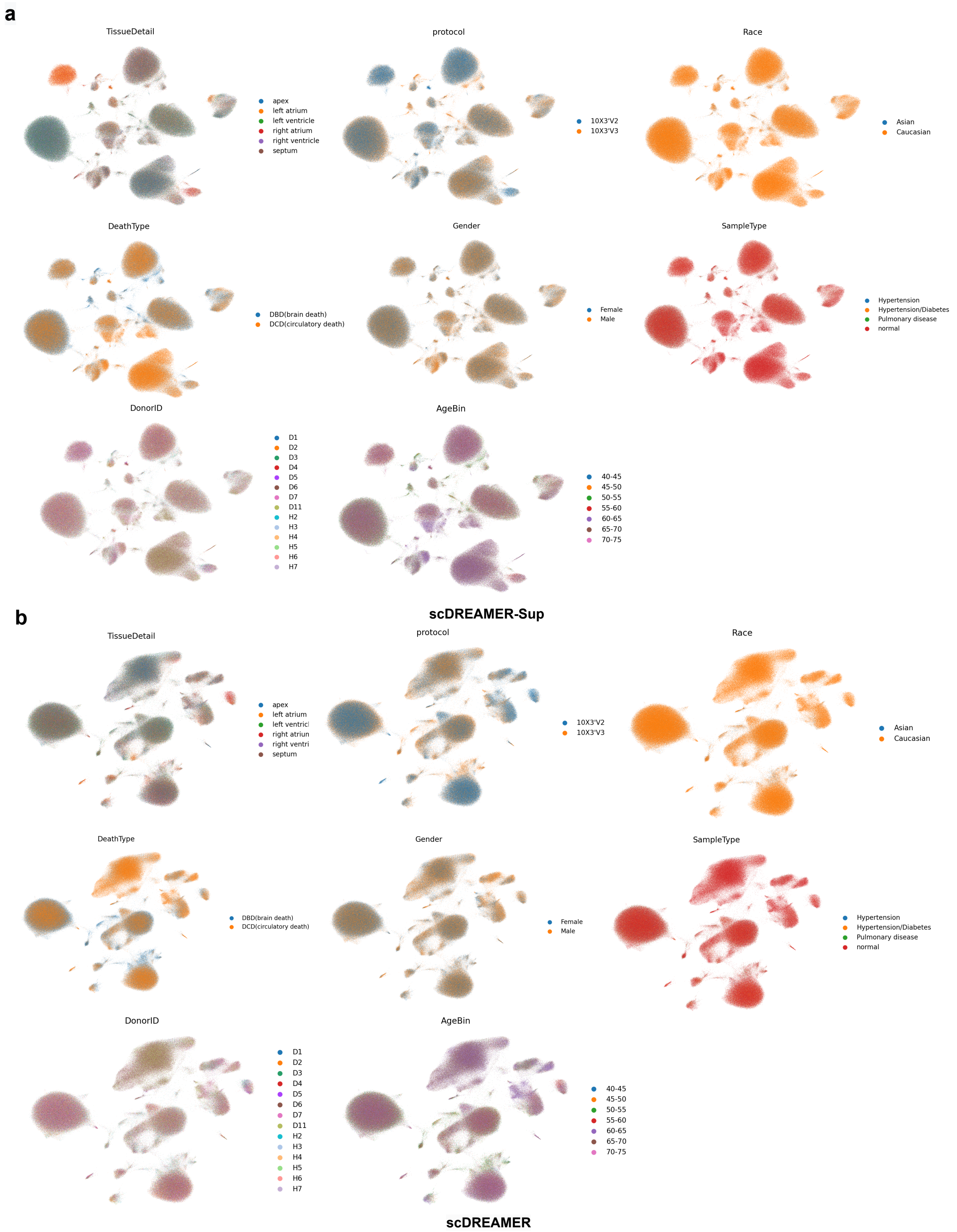

Supplementary Figure 15: Visualization of latent space embeddings inferred by scDREAMER and scDREAMER-Sup for Heart Atlas Integration annotated using tissue detail, protocol, race, death type, gender, sample type, donor and age bin: a) Visualization of scDREAMER-Sup embeddings b) Visualization of scDREAMER embeddings.

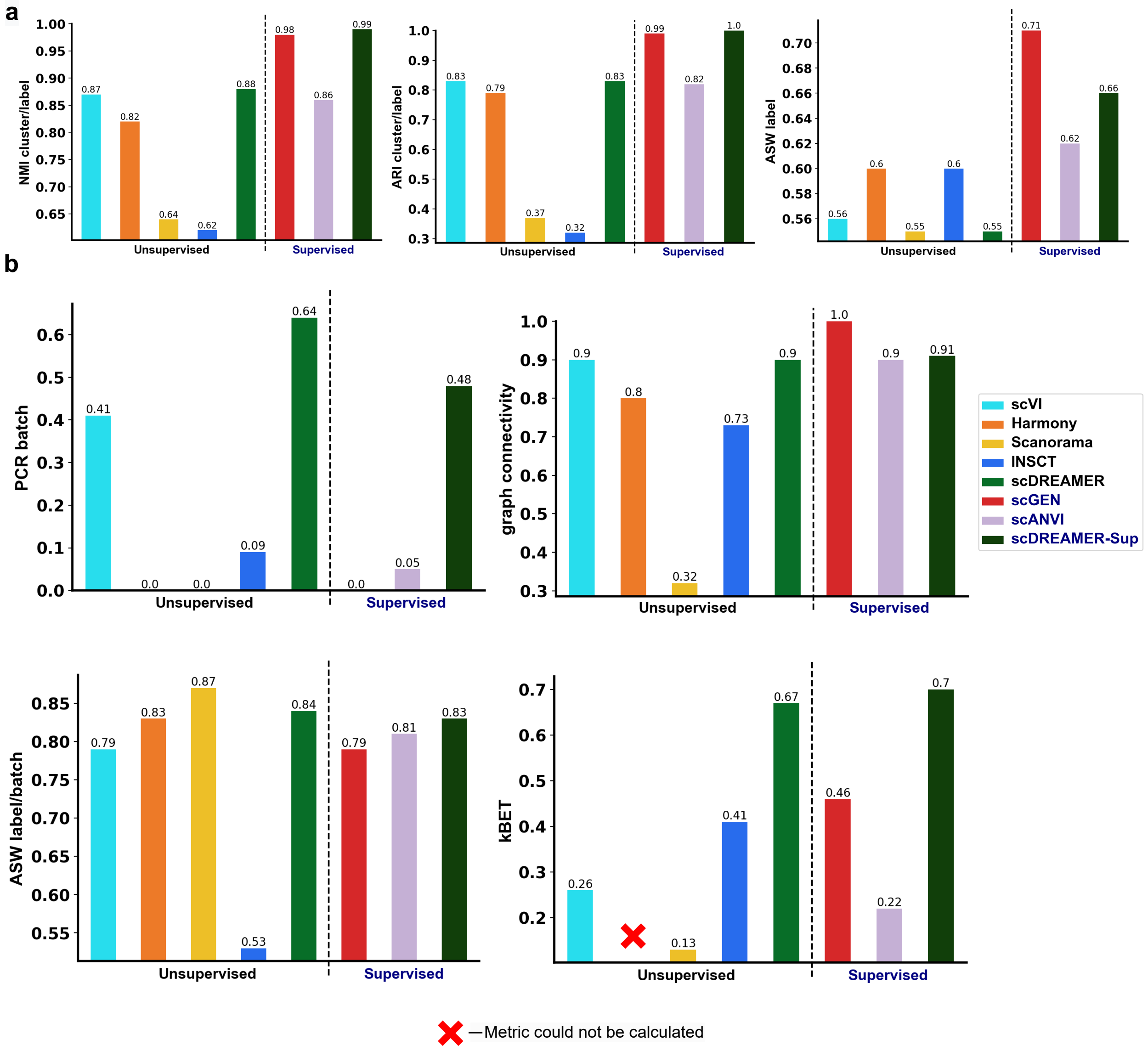

Supplementary Figure 16: Quantitative assessment of different unsupervised (scVI, Harmony, Seurat, Scanorama, INSCT, LIGER, iMAP, scDML, scDREAMER) and supervised (scGEN, scANVI, and scDREAMER-Sup) integration methods a) Comparison of bio-conservation metrics i.e. NMI, ARI and ASW. b) Comparison of batch-correction metrics i.e. PCR batch, graph connectivity, ASW label/batch and kBET. Source data are provided as a Source Data file.

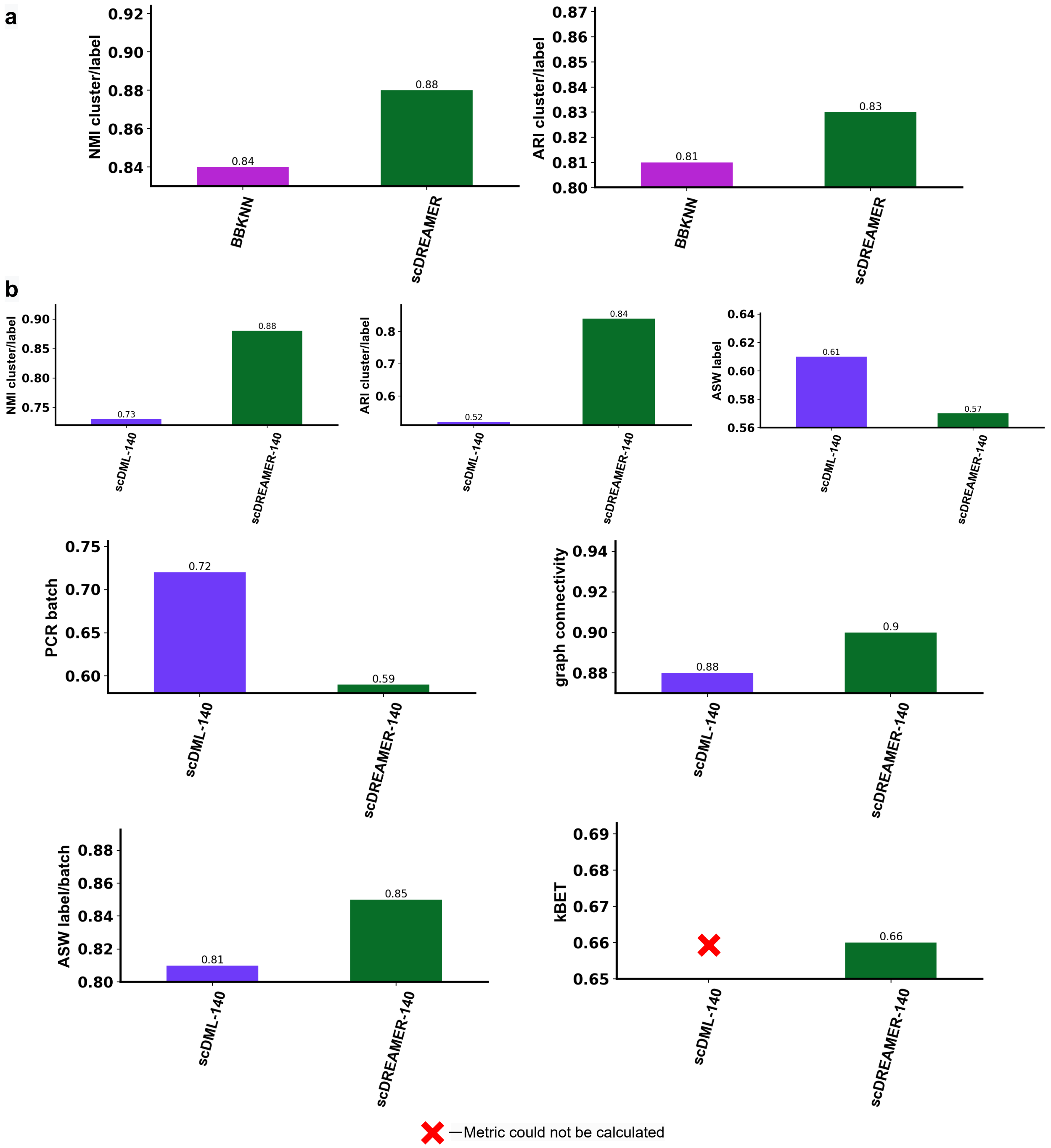

Supplementary Figure 17: a) Comparison of bio-conservation metrics i.e. NMI, ARI for BBKNN and scDREAMER for Heart Atlas Integration task. b) Quantitative assessment of scDML and scDREAMER for 140 batches of Heart Atlas Integration - comparison of bio-conservation and batch-correction metrics. Source data are provided as a Source Data file.

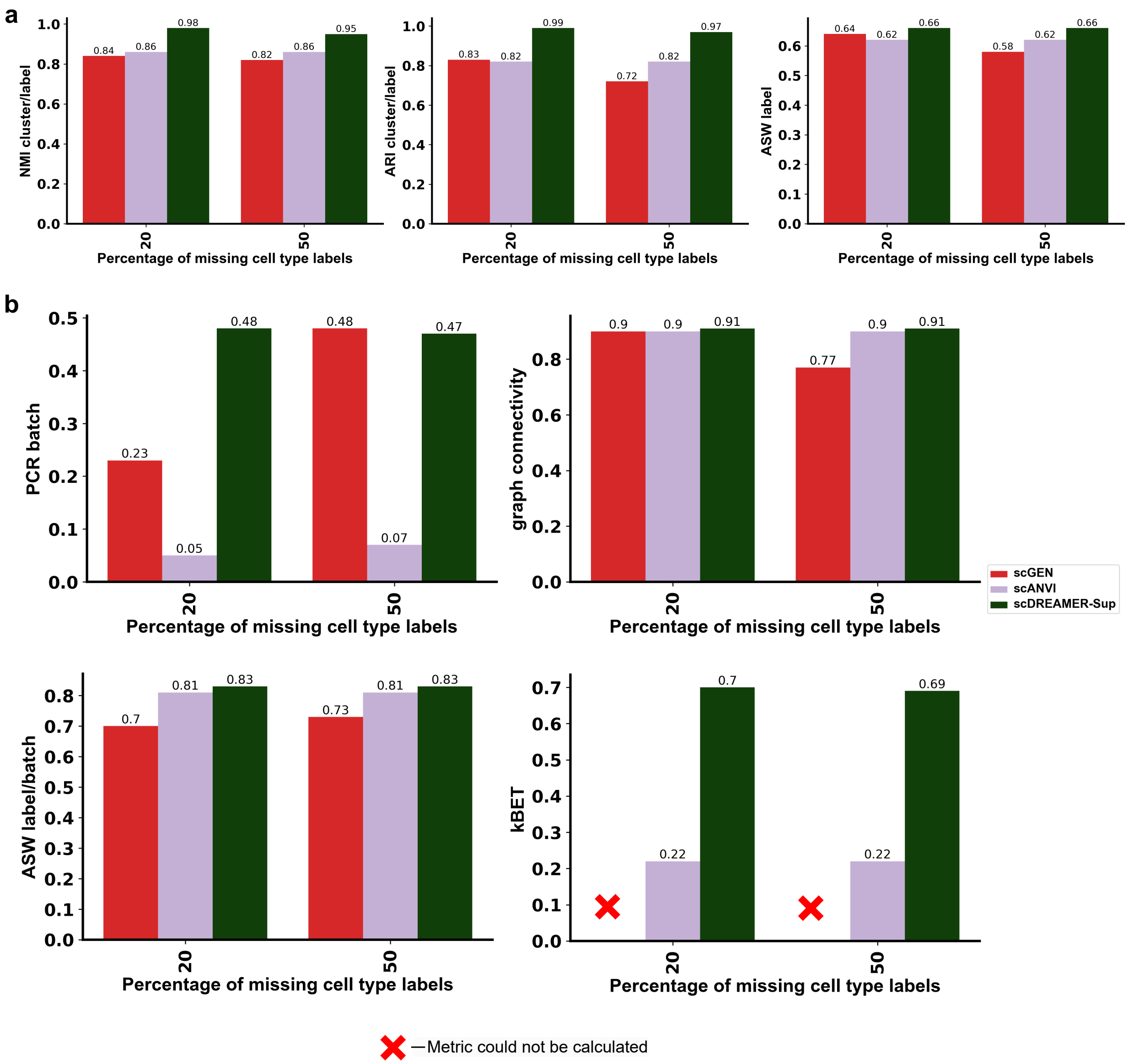

Supplementary Figure 18: Quantitative assessment of different supervised (scGEN, scANVI, and scDREAMER-Sup) integration methods for Heart Atlas Integration over different percentages of missing labels (20%, 50%). a) Comparison of bio-conservation metrics i.e. NMI, ARI and ASW. b) Comparison of batch-correction metrics i.e. PCR batch, graph connectivity, ASW label/batch and kBET. Source data are provided as a Source Data file.

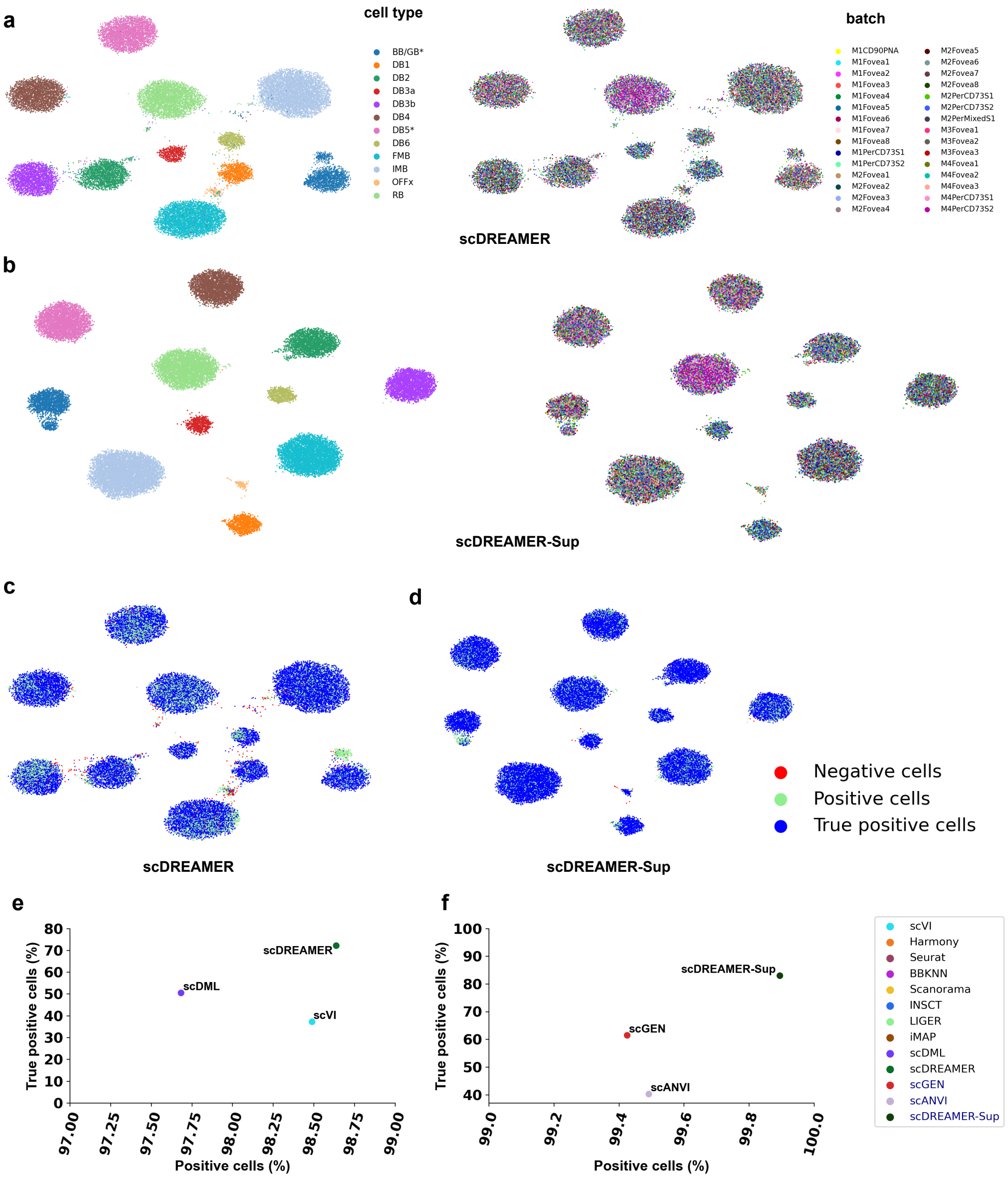

Supplementary Figure 19: **Integration of Macaque retina data.** (a) Visualization of scDREAMER's latent space embeddings after integration. Different colours denote different cell types (left) and batch information (right). (b) Visualization of scDREAMER-Sup's latent space embeddings. Qualitative assessment of batch-mixing by visualization of (c) scDREAMER's latent space embeddings, and (d) scDREAMER-Sup's latent space embeddings, cells are coloured based on three categories - positive, negative and true positive. (e) Quantitative assessment of batch-mixing of scDREAMER against scVI and scDML based on the percentage of positive vs true positive cells. (f) Quantitative assessment of batch-mixing of scDREAMER-Sup against scGEN and scANVI based on the percentage of positive vs true positive cells. Source data are provided as a Source Data file.

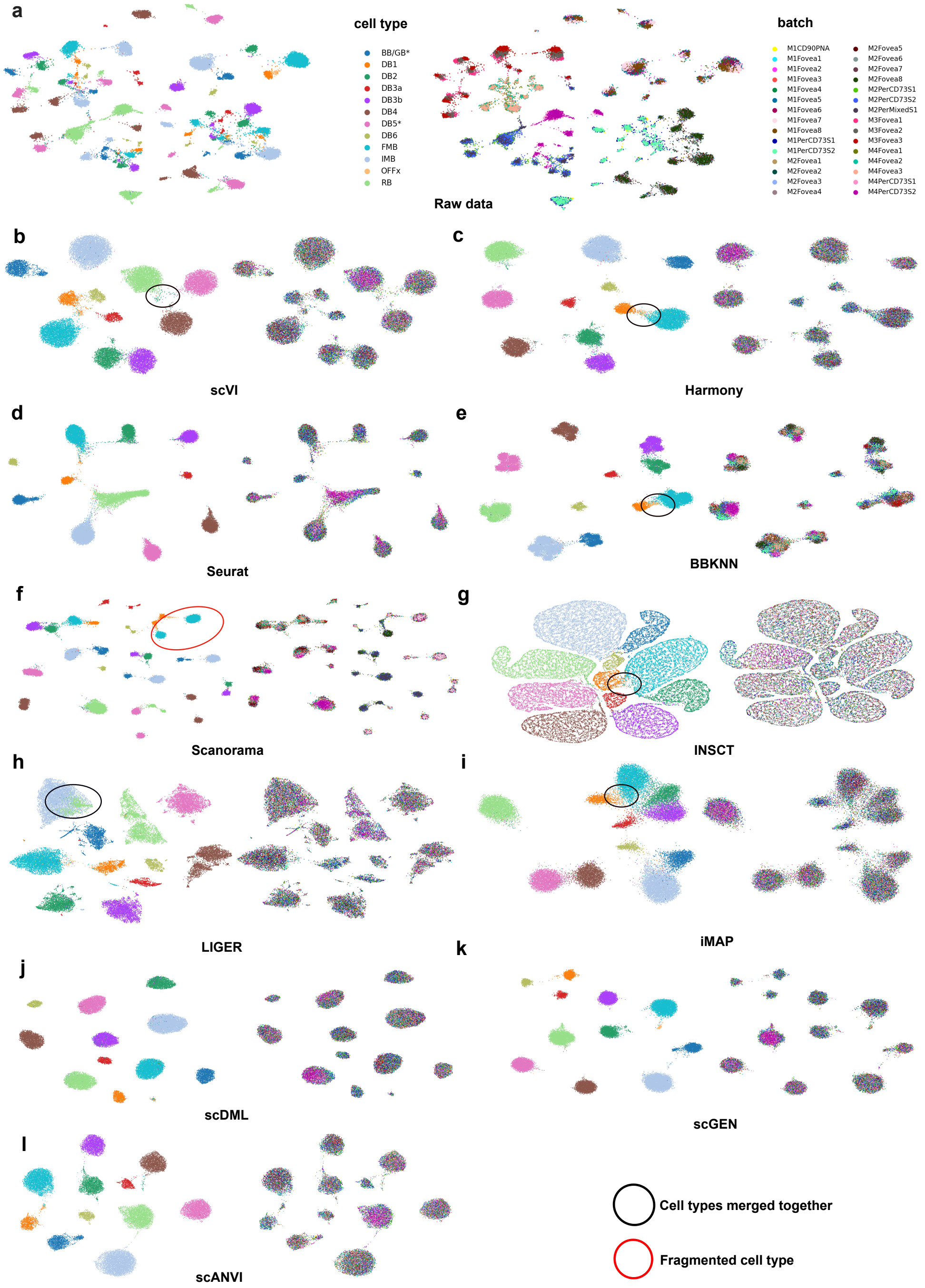

Supplementary Figure 20: Visualization of latent space embeddings for Macaque Retina Integration: a) Visualization of un-integrated macaque retina cells annotated and coloured by different cell types (left) and batch information (right). Visualization of latent space embeddings post-integration by different integration algorithms: b) scVI, c) Harmony, d) Seurat, e) BBKNN, f) Scanorama, g) INSCT, h) LIGER i), iMAP, j) scDML, k) scGEN, and l) scANVI.

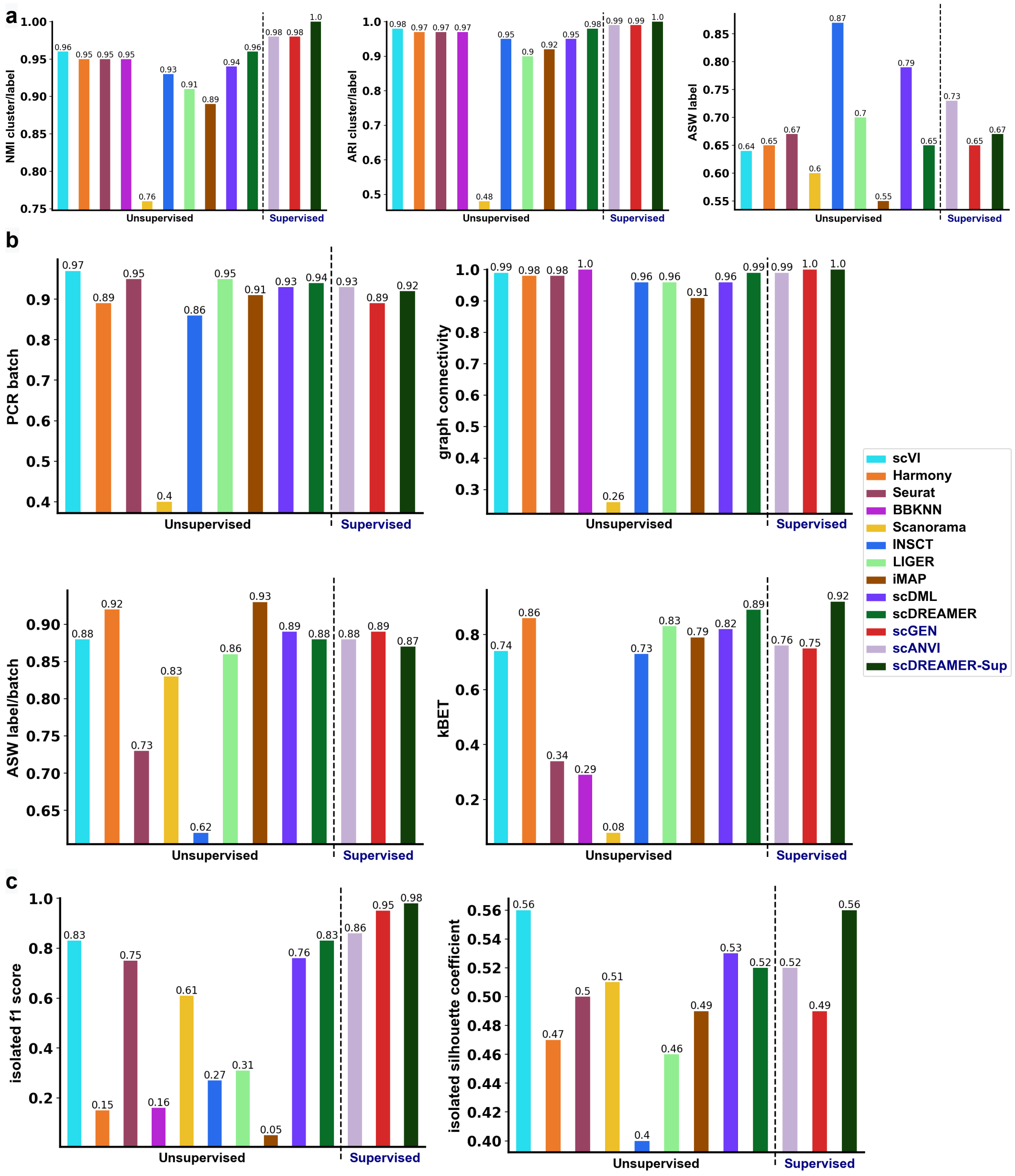

Supplementary Figure 21: Quantitative assessment of different unsupervised and supervised methods for Macaque Retina Integration: a) Comparison of bio-conservation metrics i.e. NMI, ARI, and ASW across different integration algorithms i.e. scVI, Harmony, Seurat, BBKNN, Scanorama, INSCT, LIGER, iMAP, scDML, scGEN, scANVI, scDREAMER and scDREAMER-Sup. b) Comparison of batch-correction metrics i.e. PCR batch, graph connectivity, ASW label/batch, and kBET across different integration algorithms. c) Comparison of isolated f1 score and isolated silhouette coefficient metrics across different integration algorithms. Source data are provided as a Source Data file.

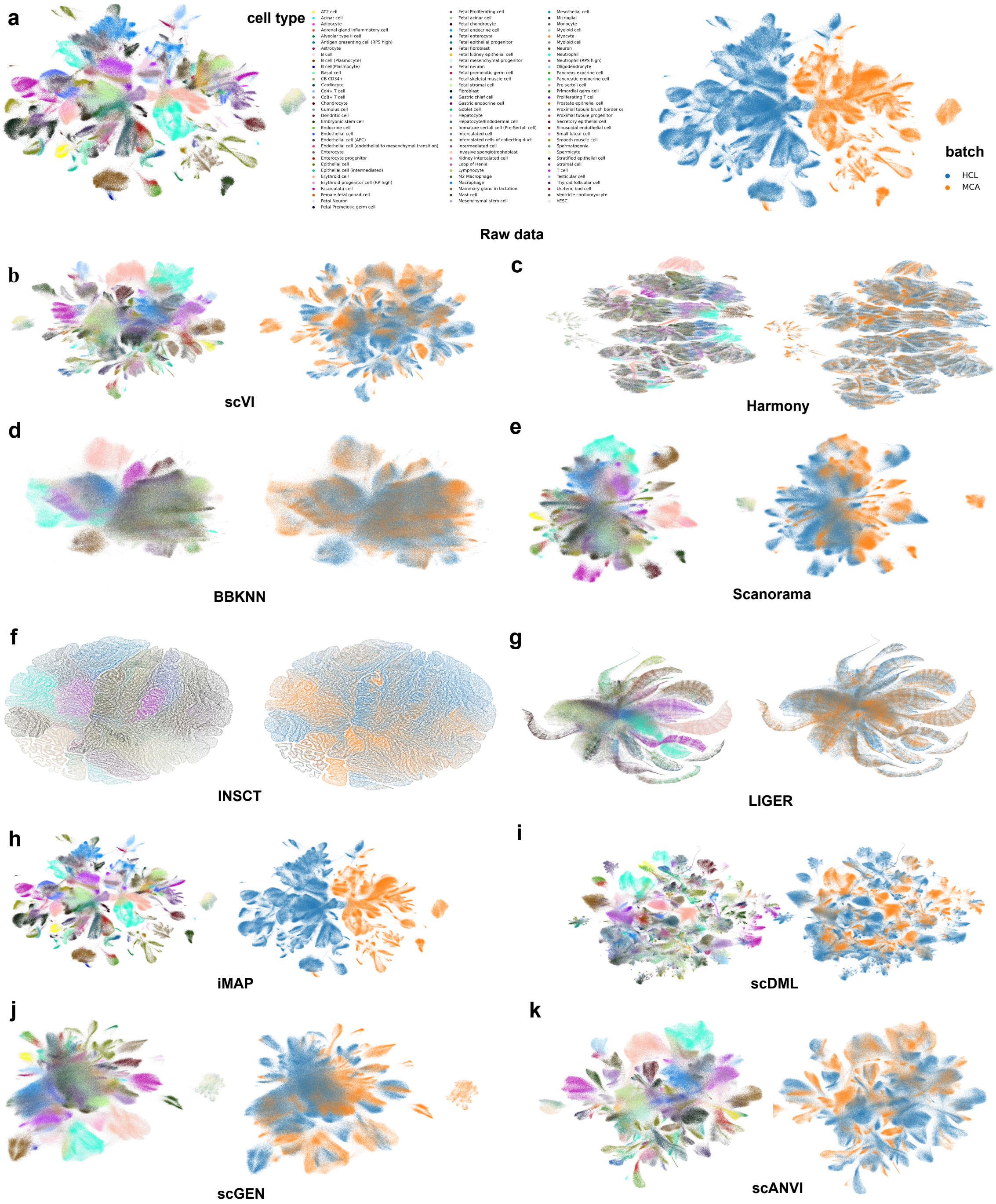

Supplementary Figure 22: Visualization of latent space embeddings for Human Mouse Integration: a) Visualization of un-integrated Human Mouse data annotated and coloured by different cell types (left) and batch information (right). The integration is performed across species i.e. human and mouse. Visualization of latent space embeddings post-integration by different integration algorithms: b) scVI, c) Harmony, d) BBKNN, e) Scanorama f) INSCT, g) LIGER h) iMAP i) scDML, j) scGEN and k) scANVI.

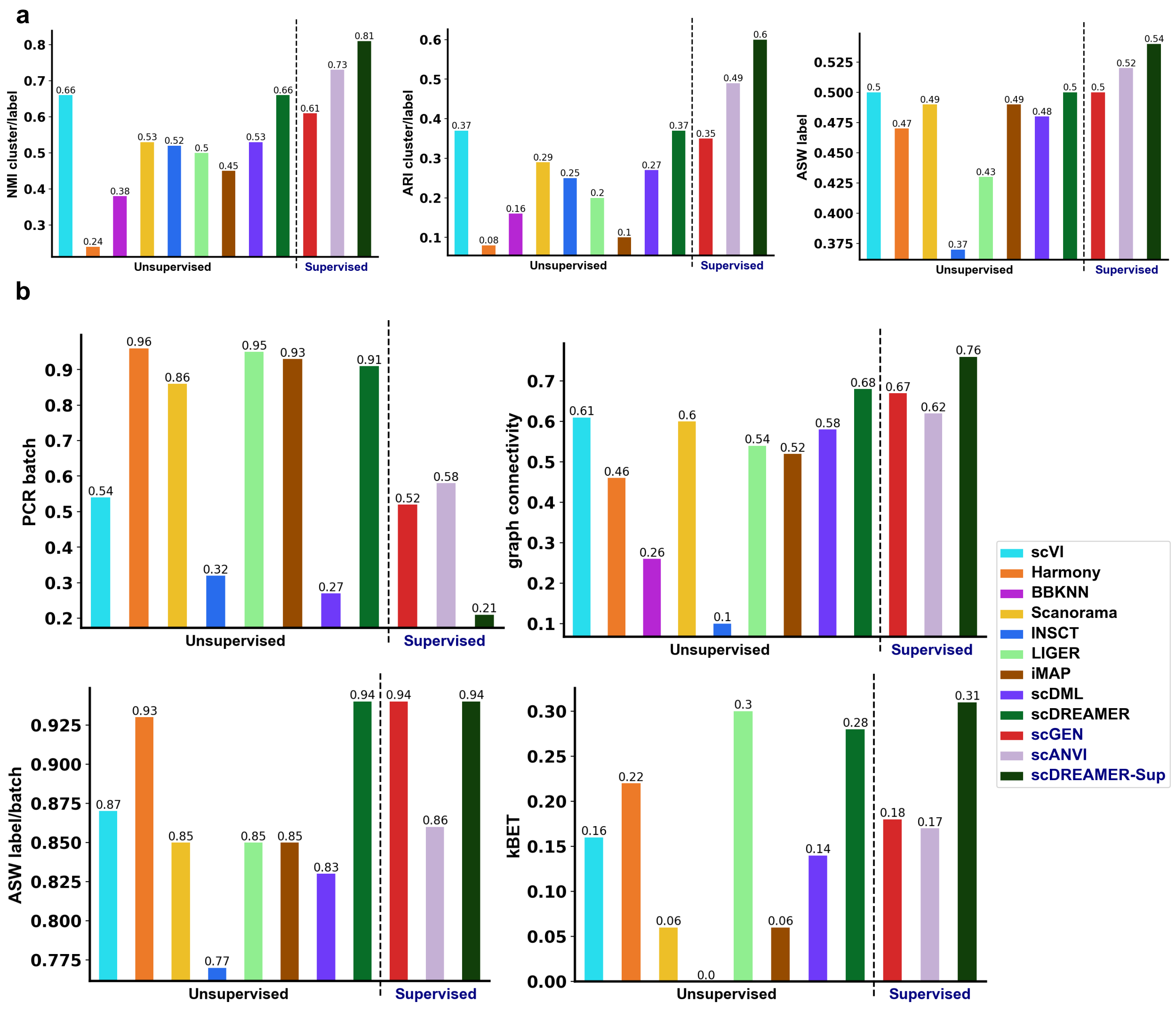

Supplementary Figure 23: Quantitative assessment of different unsupervised and supervised methods for Human Mouse Integration: a) Comparison of bio-conservation metrics i.e. NMI, ARI, and ASW across different integration algorithms i.e. scVI, Harmony, Seurat, BBKNN, Scanorama, INSCT, LIGER, iMAP, scDML , scDREAMER, scGEN, scANVI and scDREAMER-Sup. b) Comparison of batch-correction metrics i.e. PCR batch, graph connectivity, ASW label/batch, and kBET across different integration algorithms. Source data are provided as a Source Data file.

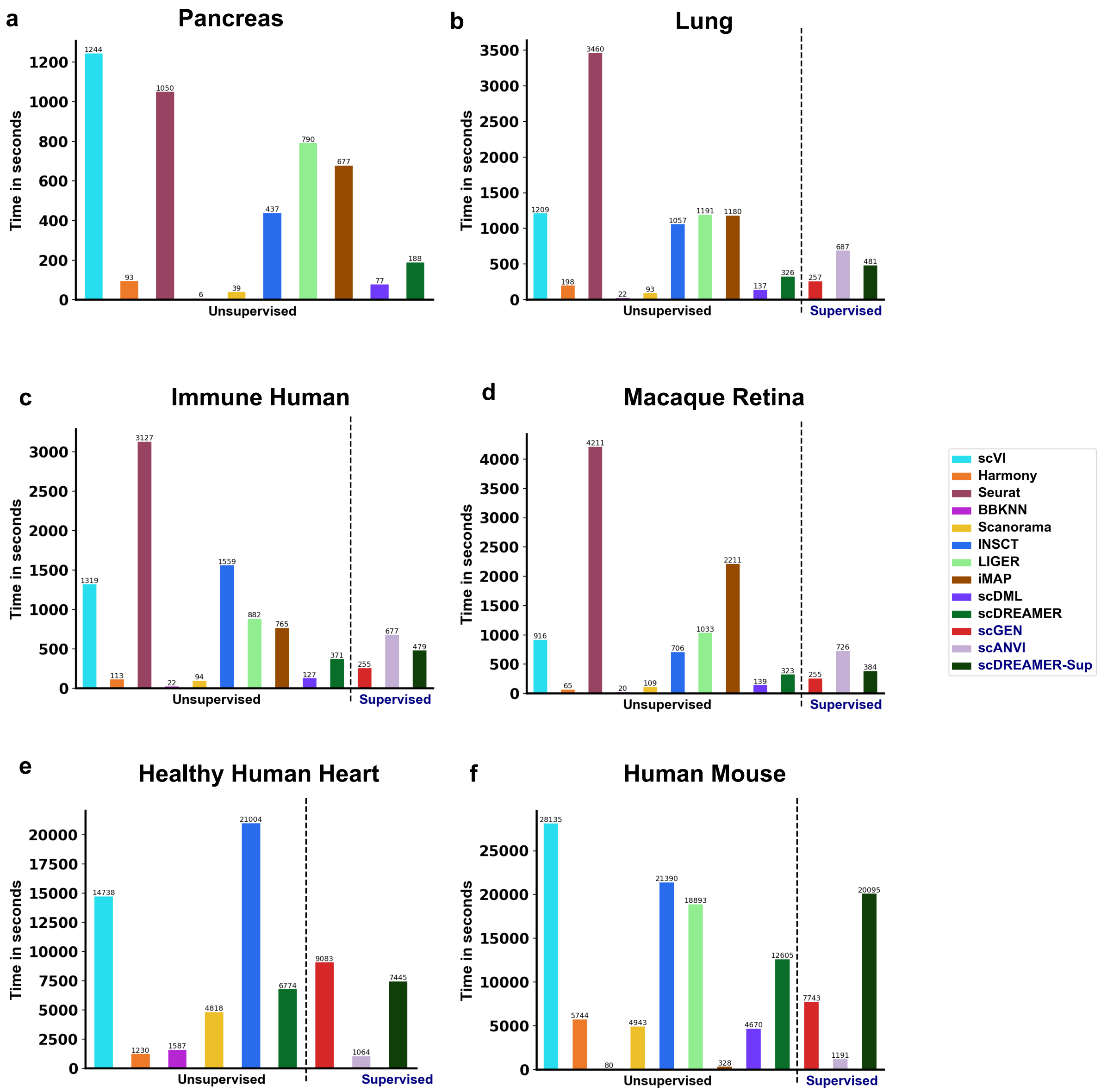

Supplementary Figure 24: Comparison of runtimes of different unsupervised and supervised methods (scVI, Harmony, Seurat, BBKNN, Scanorama, INSCT, Liger, iMAP, scDML, scDREAMER, scGEN, scANVI and scDREAMER-Sup) across different datasets - a) Pancreas dataset, b) Lung dataset, c) Human Immune dataset, d) Macaque Retina dataset, f) Healthy Heart dataset and f) Human Mouse dataset. Source data are provided as a Source Data file.

Supplementary Tables

Supplementary Table 1: Description of the datasets used for benchmarking

Dataset	Dimensions (cells * genes)	batches	cell types	Integration across	Source
Human Pancreas	16382 * 34363	9	14	Seq. protocols	link
Lung	32472 * 15148	16	17	Human donors; Laboratories	link
Immune Human	33506 * 12303	10	16	Tissue, seq. protocols	link
Macaque Retina	30302 * 18323	30	12	Macaques; Regions	link
Healthy Heart	486134 * 33638	147	11	Human donors, protocols, race, age, death and health conditions	link
Human Mouse	933704 * 4999	2	97	Species	Mouse, Human ((1))

Pancreas

The Pancreas dataset comprises of data from 9 batches with dimensions 16382*2000 (cells *genes). Here, the batches denote different sequencing techniques i.e. celseq, celseq2, fluidigmc1, inDrop1, inDrop2, inDrop3, inDrop4, smarter and smartseq2. Also, there are 14 pancreatic cell types included in the dataset, namely, acinar, activated_stellate, alpha, beta, delta, ductal, endothelial, epsilon, gamma, macrophage, mast, quiescent_stellate, schwann and t_cell. The source of the dataset is https: //figshare.com/articles/dataset/Benchmarking_atlaslevel_data_integration_in_singlecell_genomics

Lung

The Lung dataset comprises of scRNA-seq data from 16 donor batches with dimension 32472 * 15148 (cells * genes). The dataset from all batches covers 17 different cell type including B cell, Basal 1, basal 2, ciliated, dendritic cell, endothelium, fibroblast, monocytes, lymphatic, macrophage, mast cell, secretory, T/_NK cell, Type 1 cells, type 2 cells, neutrophil_CD144 _high and neutrophils_1L1R2 cells. The source of the dataset is https: //figshare.com/articles/dataset/Benchmarking_atlas−level_data_integration_in_single−cell_genomics

Immune Human

The Immune Human dataset comprises of data from 10 batches with dimensions 33506 * 12303 (cells * genes). The batches are 10X, Freytag, Oetjen_A, Oetjen_P, Oetjen_U, Sun_sample1_CS, Sun_sample2_KC, Sun_sample3_TB, Sun_sample4_TC and Villani. Also, the dataset comprises of 16 different cell types i.e.CD4+ T cells, CD8+ T cells, CD10+ B cells, VD14+ monocytes, CD16+ monocytes, CD20+ B cells, Erythrocytes, Erythroid progenitors, HSPCs, Megakaryocyte progenitors, monocyte progenitors, monocytederived dendritic cells, NK cells, NKT cells, Plasma cells and plasmacytoid dendritic cells. The source of the dataset is https://figshare.com/articles/dataset/Benchmarking_atlaslevel_data_integration_in_singlecell_genomics

Macaque Retina

The Macaque Retina dataset comprises of data from 30 batches with dimensions 30302 $\times$ 18323 (cell * genes). The data from all batches covers 12 subclusters, namely, BB/GB*, DB1, DB2, DB3a, DB3b, DB4, DB5*, DB6, FMB, IMB, OFFx and RB. The batch samples include M1CD90PNA, M1Fovea(1-8), M1PerCD73S1, M1PerCD73S2, M2Fovea(1-8), M2PerCD73S1, M2PerCD73S2, M2PerMixedS1, M3Fovea(1-3), M4Fovea(1-3), M4PerCD73S1, M4PerCD73S2. The source of the dataset is https://singlecell.broadinstitute.org/single_cell/study/

Health Heart

The Healhty Heart comprises data from 147 batches with 486134 cells. The dataset covers a total of 11 cell types, majorly covering Smooth_muscle_cells, Ventricular_Cardiomyocyte, Pericytes, Endothelial, Fibroblast, Adipocytes, Lymphoid, Myeloid, Neuronal, Atrial_Cardiomyocyte and Mesothelial cells. The batches span across samples from different donors, sequential protocols, human race, gender, age range, and death type conditions. The primary source of healthy heart data is `https://www.heartcellatlas.org/` (2). The processed data used in our study is available at `https://figshare.com/articles/dataset/Batch_Alignment_of_single-cell_transcriptomics_data_using_Deep_Metric_Learning/20499630/2` (3)

Human Mouse

The Human Mouse dataset comprises of data from 2 batches i.e. human and mouse with dimensions 933704 × 4999 (cells * genes). There are total 92 cell types, majorly covering, B cell, Acinar cells, mesothelial cells neutrophil cells, endothelial cells, epithelial cells, thyroid cells and Endocrine cells.The source of the mouse atlas data is https://figshare.com/articles/dataset/MCA_DGE_Data/5435866 and the source of human atlas data is https://figshare.com/articles/dataset/HCL_DGE_Data/7235471.

Supplementary Table 2: Ablation study on different integration tasks to analyze the contribution of the discriminator and batch classifier of scDREAMER. scDREAMER-woDis denotes scDREAMER without the discriminator and scDREAMER-woBC denotes scDREAMER without the batch classifier.

**Dataset**	**Performance Metric**	**scDREAMER**	**scDREAMER-woBC**	**scDREAMER-woDis**
Human Pancreas	Combined composite score	0.79	0.65	0.75
	Composite bio-conservation score	0.72	0.65	0.74
	Composite batch correction score	0.86	0.66	0.77
	Composite isolated label score	0.6	0.8	0.66
Lung	Combined composite score	0.58	0.5	0.56
	Composite bio-conservation score	0.45	0.47	0.42
	Composite batch correction score	0.71	0.53	0.69
	Composite isolated label score	0.76	0.67	0.74
Immune Human	Combined composite score	0.67	0.5	0.6
	Composite bio-conservation score	0.52	0.35	0.39
	Composite batch correction score	0.81	0.66	0.8
	Composite isolated label score	0.69	0.71	0.66
Healthy Heart	Combined composite score	0.69	0.53	0.7
	Composite bio-conservation score	0.48	0.49	0.51
	Composite batch correction score	0.9	0.56	0.89
Human Mouse	Combined composite score	0.78	0.73	0.79
	Composite bio-conservation score	0.67	0.72	0.7
	Composite batch correction score	0.89	0.74	0.88

Supplementary Table 3: Parameter settings used for scDREAMER training

Hyper parameters	Hyper parameter values
β, kl-scaling factor	0.001
η, learning rate (Batch-classifier and Discriminator)	{0.0007, 0.0007} {small}, {0.00001, 0.0007} {big datasets}
η_1, learning rate (Auto-encoder and Cell type classifier)	0.0002 {small}, 0.00005 - 0.0001 {big datasets}
Optimizer used	ADAM optimizer
Batch size	128
hvg, highly variable genes of sc-RNA data	2000
Epochs	$200 - 300$
z_{dim}, latent space dimensions	10
**Network**	**Architecture**
Encoder network	$hvg \longrightarrow 512 \longrightarrow z_{dim}$
Decoder network	$z_{dim} \longrightarrow 512 \longrightarrow 3 \times hvg\ (x, \mu_x, \theta_x)$
Batch-Classifier network	$z_{dim} \longrightarrow 512 \longrightarrow \lvert S \rvert$ (no. of batches)
Discriminator network	$hvg \longrightarrow 512 \longrightarrow 1$

Supplementary Table 4: Robustness of scDREAMER to learning rate parameters for Pancreas integration task

Performance Metric	η, η_1	$\eta, \eta_1 * 2$	$\eta, \eta_1/2$	$\eta_1, \eta * 2$	$\eta_1, \eta/2$
**NMI**	0.918437	0.919382	0.917982	0.917769	0.916162
**ARI**	0.950988	0.951155	0.951453	0.95068	0.949792
**ASW**	0.670919	0.672257	0.66855	0.662031	0.675863
**ASW label/batch**	0.853186	0.854505	0.852708	0.845452	0.862407
**PCR Batch**	0.92518	0.924018	0.930914	0.932537	0.918025
**graph connectivity**	0.981407	0.981025	0.979901	0.980686	0.914222
**kBET**	0.685346	0.689396	0.649419	0.673586	0.592678
**isolated f1 score**	0.103704	0.106061	0.101449	0.1	0.09589
**isolated sc width**	0.606209	0.610177	0.603815	0.654013	0.605259

Other integration methods in detail

We have compared scDREAMER against nine state-of-the-art unsupervised methods i.e. scVI (4), Scanorama (5), Harmony (6), Seurat (7), BBKNN (8), INSCT (1), LIGER (9), iMAP (10) and scDML (3). The performance of scDREAMER-Sup was compared against two state-of-the-art supervised methods - scANVI (11) and scGEN (12). More details on the methods and configuration are provided in Supplementary Table 5.

Supplementary Table 5: Configuration of the competing methods

Methods	Principle	Configuration	Github (https://github.com/)
scVI	Conditional variational autoencoder	ver: 0.7.0a5	YosefLab/scvi-tools link
Harmony	PCA + clustering-based correction	ver: 0.0.5	immunogenomics /harmony link
Seurat	CCA + Mutual nearest neighbors	ver: 4.0.6	satijalab/seurat link
BBKNN	KNN graph integration	ver: 1.5.1	Teichlab/bbknn link
Scanorama	SVD + Mutual nearest neighbors	ver: 1.7.1	brianhie/scanorama link
INSCT	Batch-aware triplet neural network	ver : 0.0.1 (Unsup.)	lkmklsmn/insct link
LIGER	Integrative non-negative matrix factorization	1.0.0	welch-lab/liger link
iMAP	Adversarial paired transfer networks	1.0.0	Svvord/iMAP link
scGEN	Supervised variational autoencoders with latent space vector arithmetics	2.1.0	theislab/scgen link
scANVI	Semi-supervised conditional VAE	0.17.3 (scvi-tools)	scverse/scvi-tools link
scDML	Deep Metric Learning	ver 1.0.0.	eleozzr/scDMLlink

Supplementary Note 1: Comparison of scDREAMER against other adversarial training approaches for scRNA-seq

DR-A (13) is a dimensionality reduction method for scRNA-seq datasets. DR-A employs an adversarial variational autoencoder consisting of two discriminators that are trained adversarially with the autoencoder. One discriminator tries to differentiate between real scRNA-seq data and the reconstructed scRNA-seq data while the other tries to discriminate the latent embeddings from an uninformed prior. The autoencoder is trained using ELBO loss and both the discriminators are trained by minimizing Bhattacharyya loss. However, DR-A does not use batch information and hence, it cannot integrate data from different batches.

MichiGAN (14) is a dimensionality reduction method that combines variational autoencoder (VAE) and generative adversarial network (GAN). MichiGAN employs an encoder, a decoder, a generator and a discriminator. First, the variational autoencoder consisting of the encoder and the decoder is trained using ELBO loss. The latent space embeddings are then used by the generator network to reproduce back the scRNA-seq data. The discriminator network then classifies between the true scRNA-seq data and the generated scRNA-seq data. Only the generator and the discriminator are trained adversarially. There is no adversarial training between variational autoencoder and generative adversarial network and both networks are trained separately.

scDGN (15) is a supervised data-integration method. It employs an encoder, a cell type/label classifier, and a batch/domain discriminator. The encoder network is trained adversarially with the batch discriminator. The cell type classifier classifies embeddings correctly into cell type anno-

tations by minimizing cross-entropy loss. The batch discriminator predicts whether a pair of latent embeddings are from the same batch or different batch by minimizing contrastive loss.

iMAP(10) is an integration method that employs one encoder and two generator networks. The encoder network extracts the low-dimensional representations of a cell. The generators are fed with the low-dimensional representations of the cell and a batch indicator. If the batch indicator is true, the original expression profile is reconstructed, if a random batch indicator is used, the generators reconstructs a fabricated expression profile. By the virtue of the adversarial generator network, the encoder is expected to capture batch-ignorant representations of the cells. The network is trained using content loss.

Adversarial deep generative models have also been developed for other computational tasks related to single-cell RNA-seq analysis such as the imputation and simulation of single-cell RNA-seq datasets. cscGAN(16) is a GAN model for the reconstruction/simulation of single-cell RNA seq data. It employs a single-cell generator (scGenerator) and a single-cell critic network. scGenerator tries to simulate single-cell RNA-seq data from standard gaussian noise and the critic network tries to classify between the true scRNA-seq data and generated scRNA-seq data. Similarly, scIGAN (17) is a GAN model for scRNA-seq imputation. To train scIGANs, the real single-cell expression profiles are first reshaped to images and fed to a generative adversarial network, wherein each cell corresponds to an image with the normalized gene expression representing the pixel. scIGANs employs a generator and discriminator networks. The generated samples are used to perform the imputation.

In all the above cases, the discriminator's adversarial training involves classification between fake and real samples. Whereas in scDREAMER, the batch-classifier is adversarially trained with the encoder so that batch classifier can learn the batch label for each cell while the encoder aims to fool the batch classifier. Moreover, scDREAMER involves two adversarial training whereas above methods rely on only one adversarial training. While scDREAMER network utilizes the batch information for training, the same is not utilized as an input by the above dimensionality reduction methods during the training. While other two batch integration methods, iMAP and scDGN utilize the batch information for training the generator networks and batch discriminator respectively, scDREAMER utilizes the batch information to train both the encoders and decoders. Moreover, the supervised version of scDREAMER, scDREAMER-Sup involves a hierarchical variational au-

toencoder which is adversarially trained with the discriminator. Such adversarial training is not employed by any of the above methods.

Supplementary References

[1] Simon, L. M., Wang, Y.-Y. & Zhao, Z. Integration of millions of transcriptomes using batch-aware triplet neural networks. *Nature Machine Intelligence* **3**, 705–715 (2021).

[2] Kanemaru, K. *et al.* Spatially resolved multiomics of human cardiac niches. *Nature* 1–10 (2023).

[3] Yu, X., Xu, X., Zhang, J. & Li, X. Batch alignment of single-cell transcriptomics data using deep metric learning. *Nature Communications* **14**, 960 (2023).

[4] Lopez, R., Regier, J., Cole, M. B., Jordan, M. I. & Yosef, N. Deep generative modeling for single-cell transcriptomics. *Nature methods* **15**, 1053–1058 (2018).

[5] Hie, B., Bryson, B. & Berger, B. Efficient integration of heterogeneous single-cell transcriptomes using scanorama. *Nature biotechnology* **37**, 685–691 (2019).

[6] Korsunsky, I. *et al.* Fast, sensitive and accurate integration of single-cell data with harmony. *Nature methods* **16**, 1289–1296 (2019).

[7] Butler, A., Hoffman, P., Smibert, P., Papalexi, E. & Satija, R. Integrating single-cell transcriptomic data across different conditions, technologies, and species. *Nature biotechnology* **36**, 411–420 (2018).

[8] Polański, K. *et al.* Bbknn: fast batch alignment of single cell transcriptomes. *Bioinformatics* **36**, 964–965 (2020).

[9] Liu, J. *et al.* Jointly defining cell types from multiple single-cell datasets using liger. *Nature protocols* **15**, 3632–3662 (2020).

[10] Wang, D. *et al.* imap: integration of multiple single-cell datasets by adversarial paired transfer networks. *Genome biology* **22**, 1–24 (2021).

[11] Xu, C. *et al.* Probabilistic harmonization and annotation of single-cell transcriptomics data with deep generative models. *Molecular systems biology* **17**, e9620 (2021).

[12] Lotfollahi, M., Wolf, F. A. & Theis, F. J. scgen predicts single-cell perturbation responses. *Nature methods* **16**, 715–721 (2019).

[13] Lin, E., Mukherjee, S. & Kannan, S. A deep adversarial variational autoencoder model for dimensionality reduction in single-cell rna sequencing analysis. *BMC bioinformatics* **21**, 1–11 (2020).

[14] Yu, H. & Welch, J. D. Michigan: sampling from disentangled representations of single-cell data using generative adversarial networks. *Genome biology* **22**, 1–26 (2021).

[15] Ge, S., Wang, H., Alavi, A., Xing, E. & Bar-Joseph, Z. Supervised adversarial alignment of single-cell rna-seq data. *Journal of Computational Biology* **28**, 501–513 (2021).

[16] Marouf, M. *et al.* Realistic in silico generation and augmentation of single-cell rna-seq data using generative adversarial networks. *Nature communications* **11**, 1–12 (2020).

[17] Xu, Y. *et al.* scigans: single-cell rna-seq imputation using generative adversarial networks. *Nucleic acids research* **48**, e85–e85 (2020).
