# Supplementary material for: scDREAMER for atlas-level integration of single-cell datasets using deep generative model paired with adversarial classifier
Source: Nat Commun. 2023 Nov 27;14:7781. doi: 10.1038/s41467-023-43590-8 (PMC10682386; doi:10.1038/s41467-023-43590-8)
Supplement: Supplementary file 3 — Reporting Summary [file 41467_2023_43590_MOESM3_ESM.pdf]

nature portfolio

Corresponding author(s): Hamim Zafar

Last updated by author(s): Oct 25, 2023

Reporting Summary

Nature Portfolio wishes to improve the reproducibility of the work that we publish. This form provides structure for consistency and transparency in reporting. For further information on Nature Portfolio policies, see our Editorial Policies and the Editorial Policy Checklist.

Statistics

For all statistical analyses, confirm that the following items are present in the figure legend, table legend, main text, or Methods section.

n/a	Confirmed	
☐	☒	The exact sample size (*n*) for each experimental group/condition, given as a discrete number and unit of measurement
☒	☐	A statement on whether measurements were taken from distinct samples or whether the same sample was measured repeatedly
☒	☐	The statistical test(s) used AND whether they are one- or two-sided<br>*Only common tests should be described solely by name; describe more complex techniques in the Methods section.*
☒	☐	A description of all covariates tested
☒	☐	A description of any assumptions or corrections, such as tests of normality and adjustment for multiple comparisons
☐	☒	A full description of the statistical parameters including central tendency (e.g. means) or other basic estimates (e.g. regression coefficient) AND variation (e.g. standard deviation) or associated estimates of uncertainty (e.g. confidence intervals)
☒	☐	For null hypothesis testing, the test statistic (e.g. *F*, *t*, *r*) with confidence intervals, effect sizes, degrees of freedom and *P* value noted<br>*Give P values as exact values whenever suitable.*
☒	☐	For Bayesian analysis, information on the choice of priors and Markov chain Monte Carlo settings
☒	☐	For hierarchical and complex designs, identification of the appropriate level for tests and full reporting of outcomes
☒	☐	Estimates of effect sizes (e.g. Cohen's *d*, Pearson's *r*), indicating how they were calculated

Our web collection on statistics for biologists contains articles on many of the points above.

Software and code

Policy information about availability of computer code

Data collection	The datasets were downloaded from GEO and other publicly available links as mentioned in the 'Data Availability' section of our manuscript. The details of the datasets are also presented in the 'Data' section below.
Data analysis	We wrote the scDREAMER tool and visualization of cellular embeddings script (based on scanpy package) and script for generating the figures. The code for performance benchmarking was adopted from PMID: 34949812. The source code and usage tutorial for scDREAMER are freely available at https://github.com/Zafar-Lab/scDREAMER, the code has also been deposited via Zenodo (https://doi.org/10.5281/zenodo.10021620). All analysis and results presented in the manuscript are available at https://github.com/Zafar-Lab/scDREAMER-reproducibility, which have also been deposited via Zenodo (https://doi.org/10.5281/zenodo.10021936).

For manuscripts utilizing custom algorithms or software that are central to the research but not yet described in published literature, software must be made available to editors and reviewers. We strongly encourage code deposition in a community repository (e.g. GitHub). See the Nature Portfolio guidelines for submitting code & software for further information.

Data

Policy information about availability of data

All manuscripts must include a data availability statement. This statement should provide the following information, where applicable:

- Accession codes, unique identifiers, or web links for publicly available datasets
- A description of any restrictions on data availability
- For clinical datasets or third party data, please ensure that the statement adheres to our policy

We did not collect any new data for the paper. All data used is referenced and available at the source.

All datasets used in this study are publicly available. The human pancreas data used in this study are available in the GEO database under accession codes GSE81076 (https://www.ncbi.nlm.nih.gov/geo/query/acc.cgi?acc=GSE81076), GSE85241 (https://www.ncbi.nlm.nih.gov/geo/query/acc.cgi?acc=GSE85241), GSE86469 (https://www.ncbi.nlm.nih.gov/geo/query/acc.cgi?acc=GSE86469), GSE84133 (https://www.ncbi.nlm.nih.gov/geo/query/acc.cgi?acc=GSE84133), GSE81608 (https://www.ncbi.nlm.nih.gov/geo/query/acc.cgi?acc=GSE81608) and the ArrayExpress database under accession code E-MTAB-5061 (https://www.ebi.ac.uk/biostudies/arrayexpress/studies/E-MTAB-5061). The lung atlas data is available in the GEO database under accession code GSE130148 (https://www.ncbi.nlm.nih.gov/geo/query/acc.cgi?acc=GSE130148). The human immune data used in this study are available in the GEO database under accession codes GSE120221 (https://www.ncbi.nlm.nih.gov/geo/query/acc.cgi?acc=GSE120221), GSE107727 (https://www.ncbi.nlm.nih.gov/geo/query/acc.cgi?acc=GSE107727), GSE115189 (https://www.ncbi.nlm.nih.gov/geo/query/acc.cgi?acc=GSE115189), GSE128066 (https://www.ncbi.nlm.nih.gov/geo/query/acc.cgi?acc=GSE128066) and GSE94820 (https://www.ncbi.nlm.nih.gov/geo/query/acc.cgi?acc=GSE94820) and in the website of 10X Genomics (PBMC10k: https://support.10xgenomics.com/single-cell-gene-expression/datasets/3.0.0/ pbmc_10k_v3).

The processed human pancreas, human immune and lung atlas datasets are available at https://figshare.com/articles/dataset/Benchmarking_atlas-level_data_integration_in_single-cell_genomics_-_integration_task_datasets_Immune_and_pancreas_/12420968. The Macaque Retina dataset is available at https://singlecell.broadinstitute.org/single_cell/study/SCP212/molecular-specification-of-retinal-cell-types-underlying-central-and-peripheral-vision-in-primates#study-download. The Healthy Heart data used in this study is available at https://www.heartcellatlas.org/. The processed Healthy Heart data used in this study can be downloaded from https://figshare.com/articles/dataset/Batch_Alignment_of_single-cell_transcriptomics_data_using_Deep_Metric_Learning/20499630/2. The Human and Mouse cell Atlas datasets used in the study are available at https://figshare.com/articles/dataset/MCA_DGE_Data/5435866 and https://figshare.com/articles/dataset/HCL_DGE_Data/7235471 respectively. The processed Human-Mouse cell atlas data can be downloaded from https://github.com/lkmklsmn/insct/tree/master/reproducibilty. All the processed datasets for the missing label experiments (for lung atlas, human immune and heart atlas tasks) are openly available at https://doi.org/10.6084/m9.figshare.24354295. The details of the experimental biological datasets used in this study are further provided in Supplementary Table 1. Source data for the different figures are provided with this paper.

Research involving human participants, their data, or biological material

Policy information about studies with human participants or human data. See also policy information about sex, gender (identity/presentation), and sexual orientation and race, ethnicity and racism.

Reporting on sex and gender	N/A
Reporting on race, ethnicity, or other socially relevant groupings	N/A
Population characteristics	N/A
Recruitment	N/A
Ethics oversight	N/A

Note that full information on the approval of the study protocol must also be provided in the manuscript.

Field-specific reporting

Please select the one below that is the best fit for your research. If you are not sure, read the appropriate sections before making your selection.

[x] Life sciences [ ] Behavioural & social sciences [ ] Ecological, evolutionary & environmental sciences

For a reference copy of the document with all sections, see nature.com/documents/nr-reporting-summary-flat.pdf

Life sciences study design

All studies must disclose on these points even when the disclosure is negative.

Sample size	We performed analysis of six different real datasets varying in the number of cells and complexity of batch effects. The analyzed datasets comprehensively cover the range of challenges in data integration task including the presence of skewed cell types among batches, nested batch effects, large number of batches, conservation of development trajectory across different batches, cross-species integration, atlas-level integration and complex multi-level batch effects. A subset of these datasets have been used in state-of-the-art benchmarking (PMID: 34949812) of data integration methods. We further designed semi-supervised integration tasks based on these datasets. Thus the collection of the used datasets and benchmarking tasks are sufficient to demonstrate the validity of the method in a wide range of integration settings.

	We did not collect any biological samples in this study. No power analysis was required as we did not need to perform any multiple group comparison with a statistical test.
Data exclusions	No data point was excluded for the datasets analyzed.
Replication	We tested scDREAMER on multiple datasets to determine its ability to work on different platforms, different biological systems and different integration settings. Given a fixed seed, scDREAMER is guaranteed to produce the same result when same dataset is used as input. The codes for reproducing the results of our experiment have been made available at https://github.com/Zafar-Lab/scDREAMER-reproducibility. Our choice of datasets, experimental settings, and analyses covers a wide range of integration scenario varying in the number of cells and complexity, reinforcing the reproducibility of the conclusions.
Randomization	No randomization was needed as no sample was collected. This study used a collection of previously published, publicly available datasets, and computational analysis of each dataset was performed independently. No randomization was required for the computational analysis of the datasets.
Blinding	Investigators were not blinded to any aspect of the study. No sample was collected or organized into experimental groups. There was no aspect of the study in which performing blinding would make sense, or in which it is previously established in the literature to perform blinding.

Reporting for specific materials, systems and methods

We require information from authors about some types of materials, experimental systems and methods used in many studies. Here, indicate whether each material, system or method listed is relevant to your study. If you are not sure if a list item applies to your research, read the appropriate section before selecting a response.

Materials & experimental systems

n/a	Involved in the study	
☒	☐	Antibodies
☒	☐	Eukaryotic cell lines
☒	☐	Palaeontology and archaeology
☒	☐	Animals and other organisms
☒	☐	Clinical data
☒	☐	Dual use research of concern
☒	☐	Plants

Methods

n/a	Involved in the study	
☒	☐	ChIP-seq
☒	☐	Flow cytometry
☒	☐	MRI-based neuroimaging
